# Fecal microbiota transplantation protects rotenone-induced Parkinson’s disease mice via suppressing inflammation mediated by the lipopolysaccharide-TLR4 signaling pathway through the microbiota-gut-brain axis

**DOI:** 10.1186/s40168-021-01107-9

**Published:** 2021-11-17

**Authors:** Zhe Zhao, Jingwen Ning, Xiu-qi Bao, Meiyu Shang, Jingwei Ma, Gen Li, Dan Zhang

**Affiliations:** State Key Laboratory of Bioactive Substrate and Function of Natural Medicine, Institute of Materia Medica, Chinese Academy of Medical Sciences and Peking Union Medical College, 1 Xian Nong Tan Street, Beijing, 100050 China

**Keywords:** Fecal microbiota transplantation, Parkinson’s disease, Rotenone-induced mouse model, Microbiota-gut-brain axis, 16S RNA sequencing

## Abstract

**Background:**

Parkinson’s disease (PD) is a prevalent neurodegenerative disorder, displaying not only well-known motor deficits but also gastrointestinal dysfunctions. Consistently, it has been increasingly evident that gut microbiota affects the communication between the gut and the brain in PD pathogenesis, known as the microbiota-gut-brain axis. As an approach to re-establishing a normal microbiota community, fecal microbiota transplantation (FMT) has exerted beneficial effects on PD in recent studies. Here, in this study, we established a chronic rotenone-induced PD mouse model to evaluate the protective effects of FMT treatment on PD and to explore the underlying mechanisms, which also proves the involvement of gut microbiota dysbiosis in PD pathogenesis via the microbiota-gut-brain axis.

**Results:**

We demonstrated that gut microbiota dysbiosis induced by rotenone administration caused gastrointestinal function impairment and poor behavioral performances in the PD mice. Moreover, 16S RNA sequencing identified the increase of bacterial genera *Akkermansia* and *Desulfovibrio* in fecal samples of rotenone-induced mice. By contrast, FMT treatment remarkably restored the gut microbial community, thus ameliorating the gastrointestinal dysfunctions and the motor deficits of the PD mice. Further experiments revealed that FMT administration alleviated intestinal inflammation and barrier destruction, thus reducing the levels of systemic inflammation. Subsequently, FMT treatment attenuated blood-brain barrier (BBB) impairment and suppressed neuroinflammation in the substantia nigra (SN), which further decreased the damage of dopaminergic neurons. Additional mechanistic investigation discovered that FMT treatment reduced lipopolysaccharide (LPS) levels in the colon, the serum, and the SN, thereafter suppressing the TLR4/MyD88/NF-κB signaling pathway and its downstream pro-inflammatory products both in the SN and the colon.

**Conclusions:**

Our current study demonstrates that FMT treatment can correct the gut microbiota dysbiosis and ameliorate the rotenone-induced PD mouse model, in which suppression of the inflammation mediated by the LPS-TLR4 signaling pathway both in the gut and the brain possibly plays a significant role. Further, we prove that rotenone-induced microbiota dysbiosis is involved in the genesis of PD via the microbiota-gut-brain axis.

**Video abstract**

**Supplementary Information:**

The online version contains supplementary material available at 10.1186/s40168-021-01107-9.

## Background

Parkinson’s disease (PD) is one of the most common neurodegenerative diseases with the average prevalence of 3.9‰ and 1–2‰ respectively in China and worldwide [1, 2]. PD patients are presented with canonical motor symptoms including slowness of movement, rigidity, resting tremors, and postural instability [3] whereas some premotor gastrointestinal (GI) dysfunctions like constipation, dysphagia, nausea, vomiting, and weight loss also frequently affect patients with PD [4, 5]. The dopaminergic neuronal death in the substantia nigra (SN) and the aggregation of α-synuclein (α-syn) in the remaining neurons are two dominant pathological hallmarks of PD which contribute to the occurrence of motor symptoms in PD patients [6].

In consistence with the presentation of GI dysfunctions which precede the classical motor symptoms in PD patients, it becomes increasingly evident that the influence of microbiota community alterations on the communications between the brain and the gut is also involved in the development of PD, known as the microbiota-gut-brain axis [7]. In healthy individuals, the stable gut microbiota composition plays a critical role in sustaining the balance of intestinal barrier integrity and inflammation, thus positively regulating brain development and behavior through the microbiota-gut-brain axis [8–10]. However, many pathological alterations of microbial taxon abundances have been reported in PD patients. For example, elevated abundances of genera *Akkermansia*, *Bifidobacterium*, and *Lactobacillus* as well as decreased levels of *Blautia* and *Faecalibacterium* in PD patients have been consistently reproduced by several studies [11–14]. In addition, the infection of *Helicobacter pylori* (Hp) has also been reported to be associated with PD [15]. Moreover, healthy mice receiving gut microbiota transplantation from PD diseased donors showed motor dysfunctions in some research [16–18]. Collectively, these reports suggest that gut microbiota dysbiosis plays a pivotal role in the pathogenesis of PD. However, the underlying mechanisms remain further exploration.

During the development of PD, gut microbiota dysbiosis can not only trigger the chronic inflammatory state of intestinal epithelium but also increase neuroinflammation through the microbiota-gut-brain axis [19, 20]. The resident inflammation in the GI tracts associated with microbiota dysbiosis may lead to the disruption of intestinal barrier integrity and the increase of its permeability, known as leaky gut [21, 22]. Then, pro-inflammatory microbial products like lipopolysaccharide (LPS) along with cytokines make their way across the damaged barrier into the circulation, thus causing systemic inflammation [8, 23]. Thereafter, these pro-inflammatory molecules in the systemic circulation may induce the destruction of the blood-brain barrier (BBB), allowing the entrance of LPS and inflammatory cytokines into the SN. As a result, neuroinflammation and cell death of dopaminergic neurons can occur in the SN [20, 24, 25]. During the whole process, LPS activates immune cells both in the intestinal tracts as well as the SN by interacting with the Toll-like receptor 4 (TLR4) on their surfaces [26, 27]. The specific mechanisms of the microbiota-gut-brain axis in PD pathogenesis still need more research to discover novel therapeutic targets for PD treatment.

Targeted at gut microbiota dysbiosis, fecal microbiota transplantation (FMT) has been emerging as a promising therapy in various diseases, such as *Clostridioides difficile* infection (CDI), and metabolic syndrome [28, 29]. It is a process of introducing normal fecal microbiota from healthy donors into the GI tracts of patients to re-establish a stable gut microbial environment, which may ameliorate the progression or the symptoms of disorders by regulating gut microbiota-associated pathways. It is also reported that FMT treatment has shown beneficial effects in several neurological disorders, like autism spectrum disorder (ASD) and multiple sclerosis (MS) [30]. In addition, one case report suggested that FMT can protect the GI dysfunctions and motor disorders in a PD patient [31]. Moreover, Sun et al. [18] showed that FMT administration alleviated a MPTP-induced PD mouse model by reducing gut microbiota dysbiosis and neuroinflammation. However, the evidence of FMT treatment for PD is still limited. More investigations are needed to evaluate its efficacy and to explore the underlying mechanisms. To further assess the protective effects of FMT treatment on PD, we established a chronic PD mouse model by rotenone and then treated the mice with FMT in the current study. Rotenone, a classic insecticide, has shown great ability of mimicking the clinical and pathological manifestations of PD [32–36]. Besides, multiple studies have also proven that the pathogenesis of the chronic rotenone-induced PD mouse model is closely related to gut microbiota dysbiosis [37–39].

Accordingly, we hypothesized that gut microbiota dysbiosis induced by rotenone exposure plays a significant role in the genesis of PD and FMT administration can prevent GI dysfunctions and motor impairments by restoring the gut microbiota in PD mice. To this end, we used a chronic rotenone-induced PD mouse model to assess whether FMT treatment can protect PD and to elucidate the underlying mechanisms, as well as to further explore the potential role of the microbiota-gut-axis in PD pathogenesis.

## Methods

### Animals and experimental design

Male C57BL/6J mice aged 8 weeks (20–22 g) were purchased from Beijing Vital River Laboratory Animal Technology Co. Ltd. (Beijing, China). The mice were then acclimatized (12-h light/dark cycle) under standard conditions (temperature 22 ± 2 °C, humidity 50–60%) with ad libitum access to food and water for 7 days. All the procedures were performed following the guidelines developed by the Beijing Municipal Ethics Committee for the care and use of laboratory animals. The animal experiments were approved by the Animal Care & Welfare Committee, Institute of Materia Medica, CAMS & PUMC (No. 00005402).

**Fig. 1:**
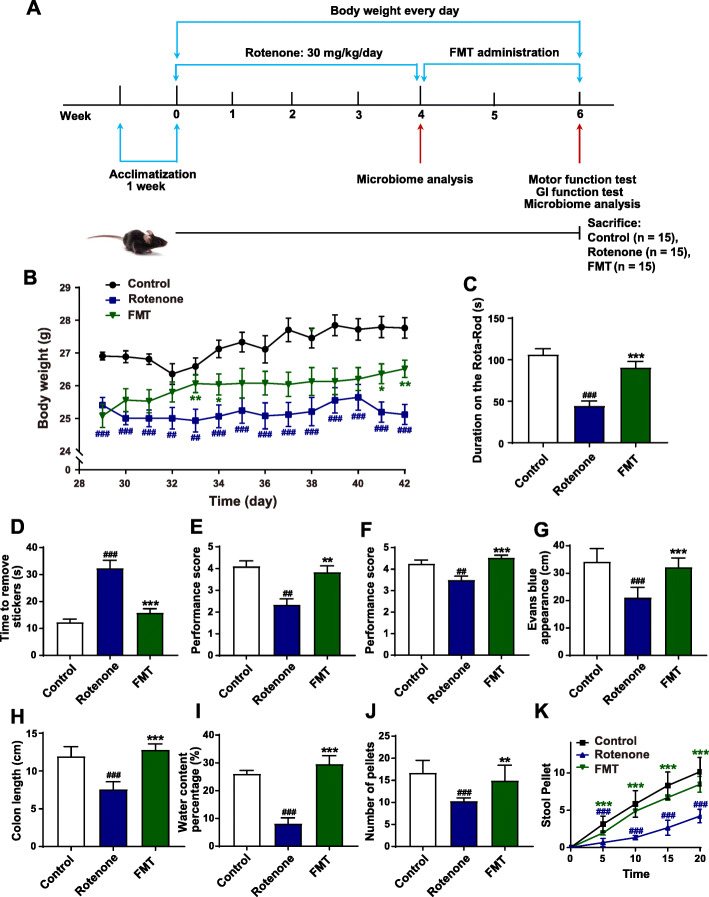
FMT treatment alleviates motor symptoms and gastrointestinal dysfunctions of the rotenone-induced PD mouse model. **A** The flow chart of animal treatments. **B** The body weights of mice from week 5 to week 6. **C** Rota-Rod test. **D** Adhesive removal test. **E** Grip strength test. **F** Pole test. **G** Intestinal transit distances. **H** Colon lengths. **I** Water percentages of fecal pellets. **J** Numbers of total fecal pellets. **K** Time course of fecal output over 20 min. For **B**–**F** and **I**–**K**, *n* = 15 for each group. For **G** and **H**, *n* = 5 replicates in each group. Data are presented as mean ± SD. ^##^*P* < 0.01, ^###^*p <* 0.001 versus the control group; *P < 0.05, **P < 0.01, ***P < 0.001 versus the rotenone group

The schematic illustration of the animal experimental design is shown as Fig. 1A. A total of 45 mice were randomly assigned into two groups: the control group (*n* = 15) and the model group (*n* = 30). In the beginning 4 weeks, the model group received the oral administration of rotenone every day. Meanwhile, the control group mice were administrated with vehicle. After 4 weeks, we randomly divided the model group mice into two groups: Rotenone group (*n* = 15) and FMT group (*n* = 15). During week 5 and 6, the mice in the FMT group were treated with FMT once per day. In the meantime, the control group and the rotenone group mice received vehicle administration once a day. These mice were daily weighed for 6 weeks. In addition, GI function tests and behavioral tests were performed at week 6. All the mice were sacrificed at week 6 for further analysis.

### Chronic rotenone model induction and FMT administration

Rotenone (Sigma-Aldrich, St. Louis, MO, USA) was dissolved in 4% carboxymethylcellulose (CMC, Sigma-Aldrich) with 1.25% chloroform (Beijing Chemical Works, Beijing, China). The fresh rotenone solution was orally administrated (30 mg/kg body weight) by gavage once a day for 4 weeks [32, 38].

For FMT administration, fresh stools were collected from the control group mice, and 600 mg of feces were immediately placed into 6 mL of sterile saline and were steeped for 1 min. Then, the dissolved feces were centrifuged at 1000 g (4 °C) for 3 min. The suspension was collected and 100 μL of bacterial suspension was then delivered to each recipient mouse via oral gavage within 10 min.

### Behavioral tests

To assess the motor function of each mouse, 4 behavioral tests were conducted with an interval of 1 h.

#### Rota-Rod test

Mice were placed on the rotarod, and then the rod revolved at a speed of 30 rpm for up to 120 s. Subsequently, the time for each mouse to fall off the rod was automatically recorded by the rotarod, which was defined as latency. Each mouse received the test for 3 times with an interval of 1 h.

#### Pole test

A 50-cm-long wooden pole, 3 cm in diameter along with a fixed wooden ball on the top of the pole was positioned in the home cage. The performance of each mouse descending the pole was scored by 6 grades, the lowest score being 0, and 5 the best. The mice received training of descending from the top to the cage before test. 3 trials were performed with an interval of 1 h.

#### Adhesive removal test

Each mouse was assigned into a clean and clear cage, and allowed 3-min free movement for acclimatization. For removal test, round adhesive labels were first placed on the front paws with slight pressure. Then, each mouse was put back into the cage, and the time for each mouse to completely remove the labels was recorded. 3 successive tests were conducted on each mouse, and an interval of 30 min between each test was allowed.

#### Grip strength test

The forepaws of the mice were allowed to grasp a horizontal rope which was 5 mm in diameter. Then the performance of each mouse on the rope was scored from 0 to 5 according to the criteria as followed: 0, falling off the rope in 10 s; 1, gripping the rope with only 1 forepaw; 2, gripping the rope with 2 forepaws; 3, gripping the rope with only 1 hind paw; 4, gripping the rope with 2 hind paws; 5, trying to escape to the end of the rope. 3 independent tests were performed for each mouse with an interval of 1 h.

### Fecal pellets output

After fasting for 2 h, each mouse was removed from their home cages and placed in a clean and transparent plastic cage for 2 h. We collected the fecal pellets and counted the numbers. Subsequently, the wet weight was obtained by weighing the fresh feces and the dry weight was weighed after 24-h drying at 85 °C. The water content percentage was calculated based on the difference in the wet and dry weights of the fecal pellets.

To evaluate the gut motor function, 20-min fecal collection was performed. Each mouse was assigned into a clean and transparent plastic cage. The numbers of stool pellets were recorded every 5 min for 20 min.

### Intestinal transit distance and colon length measurement

30 min before the sacrifice, mice were orally treated with 0.3 mL 2.5% Evans blue (Sigma-Aldrich) dissolved in 1.5% CMC-Na (Sigma-Aldrich) to detect the intestinal transit distance. Then, the distance from the pylorus to the farthest end was recorded, which was defined as intestinal transit distance [40]. In addition, the colon length between the end of the cecum and the anus was measured.

### Immunofluorescence staining

Five mice randomly chosen from each group were well sedated, and then they were transcardially well-perfused with 0.9% saline infusion followed by 4% paraformaldehyde in 0.1 M phosphate buffer. The tissues of the colons and the brains were dissected and immediately placed in 4% paraformaldehyde for 24 h, and next in 4% paraformaldehyde containing 30% sucrose. The brain and the colon tissues were embedded in paraffin and sliced into 5-μm-thick slides. The immunofluorescence staining was conducted as described in our previous study [41]. Briefly, the slides went through antigen retrieval using sodium citrate solution (pH 6.0). After being blocked with 3% bovine serum albumin (Servicebio, Wuhan, China), the slides were incubated with primary antibodies at 4 °C overnight. Primary antibodies included: anti-tyrosine hydroxylase (TH, 1:1000, Servicebio), anti-α-synuclein (1:250, Invitrogen, Waltham, MA, USA), anti-ionized calcium-binding adapter molecule 1 (Iba-1, 1:1000, Servicebio), anti-ZO-1 (1:100, Thermo Fisher Scientific, Waltham, MA, USA), anti-TLR4 (1:50, Santa Cruz Biotechnology, California, USA), anti-glial fibrillary acidic protein (GFAP, 1:1000, Servicebio) antibodies. Then, the appropriate secondary antibodies, including FITC-labeled goat anti-mouse (1:400, Servicebio), goat anti-rabbit IgG-CY3 (1:300, Servicebio), and goat anti-mouse IgG-CY3 (1:300, Servicebio) were used to detect the corresponding primary antibodies. DAPI solution was used to detect nuclei. The representative images were captured by a fluorescence microscope (Nikon Eclipse C1, Tokyo, Japan). The numbers of positive cells were analyzed by Image Pro Plus 6.0 software. Each section was calculated based on 5 randomly chosen fields.

### Hematoxylin and eosin staining

Five mice from each group were randomly chosen. The sliced colon slides of each mouse embedded in paraffin were stained with hematoxylin and eosin (H&E) following the descriptions in our previous research [41]. The histological score was blindly assessed by 2 researchers. Tissue damage was scored as the following criteria [42]: 0, no damage; 1, lymphoepithelial lesions; 2, focal ulceration or surface mucosal erosion; 3, broad mucosal damage involving deeper structures of the intestinal wall. Inflammatory cell infiltration score was evaluated according to the criteria below [42]: 0, few inflammatory cells in the lamina propria; 1, increased infiltration of inflammatory cells into the lamina propria; 2, the group of inflammatory cells infiltrating into the submucosa; 3, transmural infiltration of inflammatory cells. Then, the histological score was determined by combining the scores of tissue damage and inflammatory cell infiltration. Each section was calculated based on 5 randomly chosen fields.

### Transmission electron microscopy

The mice were well anesthetized and perfused with 0.9% saline, and both brain and colon tissues were dissected. Each sample was cut into cubes of 1 mm^3^ in size and placed into fixative at 4 °C for 4 hours. Then, the tissues were fixed in 1% osmium tetroxide at room temperature for 2 h, followed by dehydration using gradient alcohol. Subsequently, the tissues were embedded in resin and cured by baking at 60 °C for 48 h. Later, ultra-thin sections (60 nm) were cut by ultramicrotome. Finally, a transmission electron microscope (HITACHI, HT7700, Japan) was utilized to observe the ultrastructure of tight junctions both in the SN and the colon.

### Bacterial translocation detection

At sacrifice, the samples of spleen, liver, and mesenteric lymph nodes (MLNs) were aseptically collected from each mouse. The samples were smashed and suspended in 0.9% sterile saline at a ratio of 1:9, which meant that 0.1-g tissue samples were lysed with 0.9 mL 0.9% sterile saline. Then, 100 μL of the suspension was plated on a Lysogeny Broth agar plate. The numbers of colony-forming unit (CFU) were counted and analyzed after aerobic incubation at 37 °C for 24 h.

### In vivo intestinal permeability assay of FD4

The intestinal barrier integrity was evaluated by the in vivo permeability assay using fluorescein isothiocyanate-dextran (FITC-dextran, MW: 4 kDa, FD4, Sigma-Aldrich). Briefly, mice were maintained without food for 4 h and then they were orally treated with FD4 at 0.6 mg/g body weight. After another 4 h, mice were euthanized and exsanguinated by retro-orbital puncture. Immediately, serum fluorescence intensity was detected by a fluorescence spectrophotometer (485/525 nm) and FD4 concentration in the serum was further calculated based on a standard curve generated by serial dilutions of FD4.

### Quantitative polymerase chain reaction assay

Total RNA was extracted from the tissues by using the TransZol Up Plus RNA kit (TransGen Biotech Co., Beijing, China) following the manufacturer’s instructions. Then, the cDNA synthesis and the quantitative polymerase chain reaction assay (qPCR) of various genes were performed as described in our previous study [41] using the TransScript One-Step gDNA Removal and cDNA Synthesis SuperMix (TransGen Biotech Co.) and the TransStart Tip Green qPCR SuperMix (+ DyeI/+ DyeII) (TransGen Biotech Co.). The paired primers used for amplification are illustrated in Table 1. The qPCR amplification and detection were run in the 7900HT Fast Real-Time PCR system (Applied Biosystems, Foster City, CA, USA). Eventually, the relative expression of mRNA was analyzed using the 2^-ΔΔCt^ algorithm.

**Table 1:** Paired primers for qPCR

Gene	Primer sequence (5′-3′)	
	Forward	Reverse
*Tnf*	ACGGCATGGATCTCAAAGAC	AGATAGCAAATCGGCTGACG
*Il1b*	GCTACCTATGTCTTGCCCGT	GACCATTGCTGTTTCCTAGG
*Il6*	CTGCAAGAGACTTCCATCCAG	AGTGGTATAGACAGGTCTGTTGG
*COX2*	GAAGTGGGGGTTTAGGATCATC	CCTTTCACTTTCGGATAACCA
*Nos2*	CAGCTGGGCTGTACAAACCTT	CATTGGAAGTGAAGCGTTTCG
*Ocln*	TTACAGGCAGAACTAGACGAC	TGATGTGCGATAATTTGCTCT
*ZO1*	TTCCCAGCTTATGAAAGGGTT	TCGCTTCTTTCAGGGCACCGTA
*Cldn1*	GCCATCTACGAGGGACTG	GAGCAGGAAAGTAGGACACC
*Cldn5*	ACTGCCTTCCTGGACCACAAC	CGCCAGCACAGATTCATACACCT
*Tlr4*	AACTTCAGTGGCTGGATT	ACTAGGTTCGTCAGATTGG
*Gapdh*	ATGACTCTACCCACGGCAAG	GATCTCGCTCCTGGAAGATG

### Western blot

For total protein extraction, RIPA lysis buffer (C1055, APPLYGEN, China) with protease inhibitor cocktail (P1265, APPLYGEN, China) and phosphatase inhibitors mixture (P1260, APPLYGEN, China) was utilized to lyse the tissues. Then, the lysate was centrifuged at 12,000 g, 4 °C for 20 min to obtain the total protein. To separate cytoplasmic and nuclear proteins, the Nuclear/cytoplasmic Isolation Kit (P1201, APPLYGEN, China) was used according to the manufacturer’s instruction. As described in our previous study [43], the protein concentrations were detected by the BCA Protein Assay Kit (P1511, APPLYGEN, China) and western blot was performed using sodium dodecyl sulfate polyacrylamide gel electrophoresis (SDS-PAGE) method. The transferred membranes were incubated at 4 °C overnight with the following primary antibodies: mouse anti-TH antibody (1:1000, MAB318, Millipore), rabbit anti-tumor necrosis factor-α (TNF-α) antibody (1:500, AF7014; Affinity Biosciences, Cincinnati, OH, USA), rabbit anti-interleukin-1β (IL-1β) antibody (1:1000, ab200478; Abcam, Cambridge, MA, USA), rabbit anti-interleukin-6 (IL-6) antibody (1:1000, ab229381; Abcam), rabbit anti-inducible NO synthase (iNOS) antibody (1:1000, A0312; Abclonal, China), goat anti-cyclooxygenase 2 (COX2) antibody (1:1000, ab23672; Abcam), mouse anti-α-synuclein (1:1000, 32-8100; Invitrogen), mouse anti-TLR4 antibody (1:400, ab22048; Abcam), rabbit anti-MyD88 (1:400, ab2064; Abcam, Cambridge, MA, USA), rabbit anti-IκB-α (1:400, sc-371; Santa Cruz Biotechnology Inc., Santa Cruz, CA, USA), rabbit anti-ZO-1 antibody (1:1000, ab96587, Abcam), mouse anti phosphorylated-IκB-α (1:400, sc-8404; Santa Cruz Biotechnology Inc., Santa Cruz, CA, USA), rabbit anti-NF-κB p65 antibody (1:1000, A2547, Abclonal, China), mouse anti-claudin-1 antibody (1:1000, 2H10D10, Invitrogen), rabbit anti-occludin antibody (1:1000, 27260-1-AP, ProteinTech), mouse anti-claudin-5 antibody (1:1000, A10207, Abclonal, China). The membranes were then incubated with the corresponding secondary antibodies, including HRP anti-mouse antibody (1:2000, AS003; Abclonal), HRP anti-rabbit antibody (1:2000, AS014; Abclonal), HRP anti-goat antibody (1:2500, AS031; Abclonal) for 2 h at room temperature. The blots were visualized using LAS4000 chemiluminescence system (Fujifilm, Tokyo, Japan), and the densities were analyzed by Gel-Pro Analyzer 4.0 software.

### Enzyme-linked immunosorbent assay

The enzyme-linked immunosorbent assay (ELISA) kits used to detect the levels of mouse TNF-α, IL-1β, IL-6, LPS endotoxin, and lipopolysaccharide binding protein (LBP) were purchased from Jianglai Industrial Limited By Share Ltd, Shanghai, China. The experimental procedures were carried out according to the manufacturer’s instructions, which is similar to the descriptions in our previous study [41]. The concentrations of target proteins were determined by standard protein curves.

### Fecal DNA extraction and 16S RNA sequencing

Mice were randomly chosen from each group at week 4 and week 6 for microbiota sequencing analysis. After placing each mouse in a separate empty autoclaved cage, 6-8 fresh fecal pellets from each mouse were collected and immediately put into a sterile EP tube. All the fecal samples were instantly frozen and stored at – 80 °C for further analysis. Microbial genomic DNA was extracted from fecal samples by a QIAamp Fast DNA Stool Mini Kit (Qiagen, Germany) following the improved protocol based on the manufacturer’s instructions. The V3-V4 regions of the microbial 16S RNA were amplified with the paired primers (forward primer: 5′-CCTACGGGRSGCAGCAG-3′; reverse primer: 5′-GGACTACVVGGGTATCTAATC-3′). The following condition was used: 95 °C for 3 min, followed by 30 cycles at 98 °C for 20 s, then 58 °C for 15 s, and 72 °C for 20 s and a final extension at 72 °C for 5 min. All the quantified amplicons were pooled together at equalized concentrations for Illumina MiSeq sequencing (Illumina, Inc., CA, USA). Experiments including DNA extraction, quality assessment, library construction, and high-throughput sequencing were performed by Realbio Genomics Institute (Shanghai, China).

### Bioinformatic analysis

Sequencing data were processed and analyzed by Realbio Genomics Institute (Shanghai, China). Briefly, PANDAseq (V2.9) was used to filter the clean reads by removing the reads with the average Phred score lower than 20, removing the reads containing more than 3 ambiguous bases, and retaining the reads of 250–500 nt [44]. Then, operational taxonomic units (OTUs) were obtained using Usearch (V7.0.1090) [45] by clustering the sequences with similarities over 97%. Then, OTUs were annotated by comparing representative sequences to the Ribosomal Database Project (RDP, http://rdp.cme.msu.edu) [46, 47]. Alpha-diversity indices (Shannon index and Simpson index) were calculated by QIIME (V1.9.1) [48] and the differences among the 3 groups were analyzed by the Kruskal-Wallis test on R (V3.5.1). Beta-diversity analysis was performed using weighted UniFrac distances. Thereafter, the difference comparisons among different groups were conducted by Adonis and were displayed by the principal coordinate analysis (PCoA) method. Besides, the analysis of similarity (ANOSIM) method was also performed to compare the group differences. Linear discriminant analysis (LDA) was used for comparing the differences of microbial abundances at different taxon levels by LDA EffectSize Tools (V1.0) [49]. Then, the microbiota community structures were displayed by Graphical Phylogenetic Analysis software. The correlations between the bacterial relative abundances and other experimental results were performed using Spearman correlation analysis and were displayed by heatmap on R (V3.5.1). Eventually, the functional prediction analysis was performed by the Phylogenetic Investigation of Communities by Reconstruction of Unobserved States (PICRUSt) method to determine Kyoto Encyclopedia of Genes and Genomes (KEGG) pathways [50]. The analysis was conducted using R (V3.5.1).

### Statistical analysis

The statistical analysis was conducted by using SPSS software (version 20.0). All the data were displayed as mean ± standard deviation (SD). Statistical analysis for multiple comparisons was performed by one-way analysis of variance (ANOVA) followed by the least significant difference (LSD) post-hoc test. For pole test performance, grip strength test performance, histological score, ZO-1 integrity score, and cell numbers, the non-parametric Kruskal-Wallis test was used, followed by the Mann-Whitney *U* post-hoc test. For 2 group comparisons, the independent t test was utilized. To obtain the correlations between different experiments, Spearman correlation analysis was performed by R (V3.5.1). When the *P* value was < 0.05, the results were considered statistically significant.

## Results

### FMT treatment alleviates motor symptoms and gastrointestinal dysfunctions of the rotenone-induced PD mouse model

Weight loss, motor disorders, and GI dysfunctions commonly occur in PD animal models. To evaluate the protective effects of FMT administration on PD, we established a chronic PD mouse model by orally treating the mice with rotenone for 4 weeks. In the following 2 weeks, the rotenone-challenged mice were administrated with FMT or vehicle (Fig. 1A). During the establishment of the PD model, the rotenone group mice began to show significant weight loss from day 20 (data not shown). As illustrated in Fig. 1B, FMT treatment markedly alleviated the weight loss of rotenone-challenged mice in weeks 5 to 6. At week 6, 4 kinds of behavioral tests were performed to assess the motor functions of mice from different groups, including the Rota-Rod test for motor coordination, the adhesive removal test for sensorimotor integration, the grip strength test for muscle force of limbs, and the pole test for motor balance (Fig. 1A). The rotenone-intoxicated mice were present with significant motor disorders compared to the control group mice, including decreased time on the rod (*P* < 0.001, Fig. 1C), delayed removal from the stickers (*P* < 0.001, Fig. 1D), poorer performance of the limb muscle force (*P* < 0.01, Fig. 1E), and decreased scores of the pole test (*P* < 0.01, Fig. 1F). On the contrary, the performances of the FMT group mice improved remarkably in the Rota-Rod test (*P* < 0.001, Fig. 1C), the adhesive removal test (*P* < 0.001, Fig. 1D), the grip strength test (*P* < 0.01, Fig. 1E), and the pole test (*P* < 0.001, Fig. 1F) compared to the rotenone group.

In addition, GI dysfunctions were also measured in our study. Evans blue was applied to indicate the intestinal transit functions of mice and the colon length was measured. The rotenone group mice showed a significantly decreased intestinal transit distance (*P* < 0.001, Fig. 1G) and colon length (*P* < 0.001, Fig. 1H) compared to the control group mice. On the contrary, FMT treatment remarkably attenuated these two dysfunctions (both *P* < 0.001, Fig. 1G-H) induced by rotenone. Besides, fecal pellets were collected to measure the water content percentage and the colon motility. The rotenone-challenged mice showed markedly reduced fecal water content percentages (*P* < 0.001, Fig. 1I) and pellet numbers (*P* < 0.001, Fig. 1J), which were significantly elevated by FMT administration (water content percentage, *P* < 0.001; pellet number, *P* < 0.01) (Fig. 1I-J). Moreover, remarkable differences in fecal output frequencies (indicated by a 20-min fecal collection experiment) between the rotenone group mice and the control group mice were observed in the study (Fig. 1K) while FMT treatment significantly increased the fecal output frequencies of rotenone-challenged mice (Fig. 1K). Taken together, these data suggest that rotenone intoxication induces weight loss, motor disorders, and GI dysfunctions in the PD mouse model whereas FMT administration significantly attenuates these PD manifestations.

### FMT administration attenuates PD-associated histological features and inflammation in the SN and the colon of the rotenone-intoxicated mouse model

**Fig. 2:**
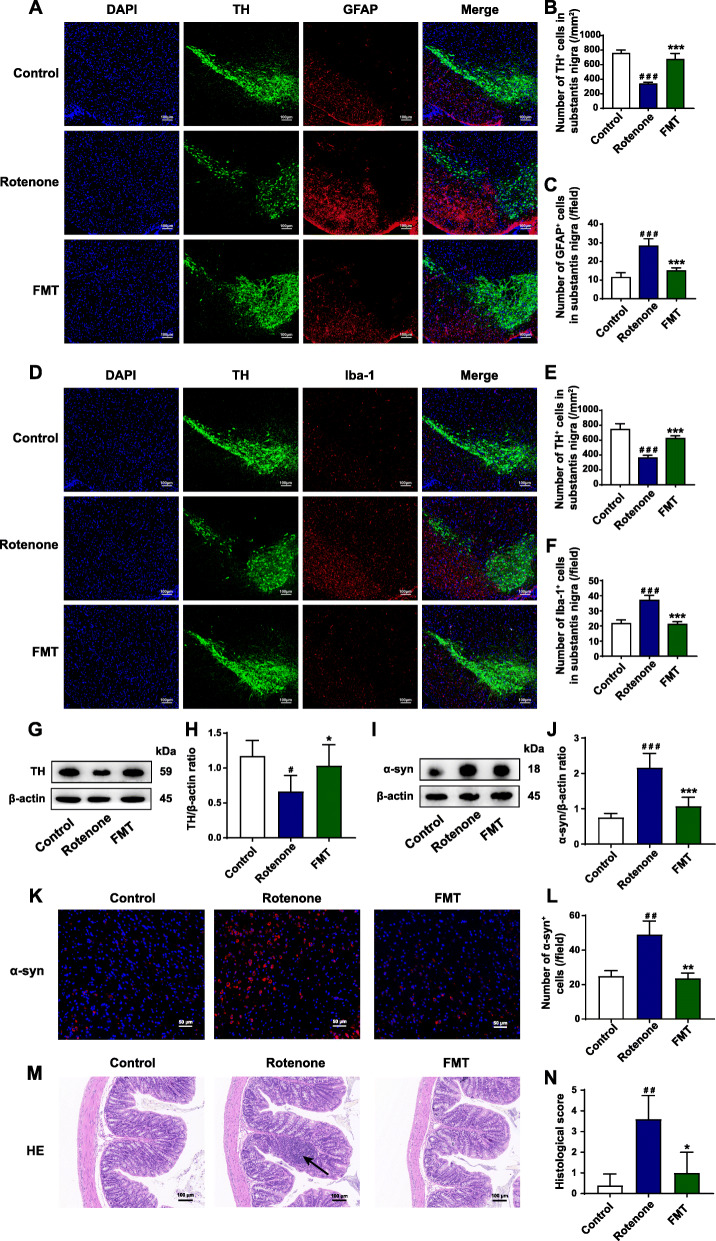
FMT administration attenuates PD-associated histological features and inflammation in the SN and the colon of the rotenone-intoxicated mouse model. **A** Representative captures of immunofluorescence in the SN of nuclei (DAPI, blue), total dopaminergic neurons (TH, green), and astrocytes (GFAP, red). **B** Numbers of TH^+^ cells in the SN. **C** Numbers of GFAP^+^ cells (activated astrocytes) in the SN. **D** Representative captures of immunofluorescence in the SN of nuclei (DAPI, blue), total dopaminergic neurons (TH, green), and microglial cells (Iba-1, red). **E** Numbers of TH^+^ cells in the SN. **F** Numbers of Iba-1^+^ cells (activated microglial cells) in the SN. **G** Representative western blot brands of TH in the midbrain containing the SN. **H** The density analysis result of TH western blot in the midbrain containing the SN. **I** Representative western blot brands of α-syn in the midbrain containing the SN. **J** The density analysis result of α-syn western blot in the midbrain containing the SN. **K** Representative captures of immunofluorescence in the SN of α-syn. **L** Statistical analysis of α-syn density in the SN. **M** Representative captures of H&E staining in the colon; the black arrow indicates inflammatory infiltration. **N** Histological scores of colons based on H&E staining. For **B** and **C**; **E** and **F**; **L**; and **N**, *n* = 5 for each group. For **H** and **J**, *n* = 4 for each group. Data are presented as mean ± SD. ^#^*P* < 0.05, ^##^*P* < 0.01, ^###^*P* < 0.001 versus the control group; *P < 0.05, **P < 0.01, ***P < 0.001 versus the rotenone group

To further explore how FMT treatment protected GI dysfunctions and motor deficits, we detected the histological characteristics and inflammation both in the SN and the colon using various experiments. It is well acknowledged that the loss of dopaminergic neurons and the accumulation of α-syn are two histological hallmarks of PD [6]. In the current study, the TH^+^ cells (dopaminergic neurons) in the SN of the rotenone group mice decreased to nearly half of the control group mice (*P* < 0.001), while FMT treatment remarkably restored the neuronal loss (*P* < 0.001) (Fig. 2A, B, D, and E). Consistently, western blot of the midbrain containing the SN validated that the expression of TH in the rotenone group remarkably decreased compared to the control group (*P* < 0.05) and this reduction was reversed by FMT administration (*P* < 0.05) (Fig. 2G, H). As another important PD histological hallmark in the SN, a significantly increased α-syn expression of the rotenone group mice was found by western blot of the midbrain containing the SN (*P* < 0.001) and immunofluorescence staining (*P* < 0.01) (Fig. 2I-L). However, FMT treatment markedly reduced the aggregation of α-syn (western blot, *P* < 0.001; immunofluorescence staining, *P* < 0.01) (Fig. 2I-L).

It has been reported that the activation of microglial cells and astrocytes may contribute to neuroinflammation and dopaminergic neuronal death in the SN during PD development [51, 52]. In our present study, we performed immunofluorescence staining of the SN region to detect the activation of glial cells, using GFAP as the marker of astrocytes, and Iba-1 as the marker of microglia. Both GFAP^+^ and Iba-1^+^ cells showed significant elevation in the SN region of the rotenone group mice (both *P* < 0.001, Fig. 2A, C, D, and F). However, FMT administration markedly attenuated the elevation of GFAP^+^ cells (rotenone group, 28 cells/field; FMT group, 15 cells/field; *P* < 0.001) and Iba-1^+^ cells (rotenone group, 37 cells/field; FMT group, 21 cells/field; *P* < 0.001) in the SN region of rotenone-intoxicated mice (Fig. 2A, C, D, and F).

In addition, immune infiltration and epithelium impairment in the colon were evaluated by H&E staining. The H&E histological scores were remarkably higher in the rotenone group than the control group (*P* < 0.01) while FMT administration reduced the scores significantly (*P* < 0.05) (Fig. 2M, N). Collectively, these results illustrate that FMT treatment significantly attenuates PD-related histological characteristics and inflammation both in the SN and the colon of rotenone-challenged mice.

### FMT treatment restores BBB and intestinal barrier impairment in the rotenone-challenged mouse model

**Fig. 3:**
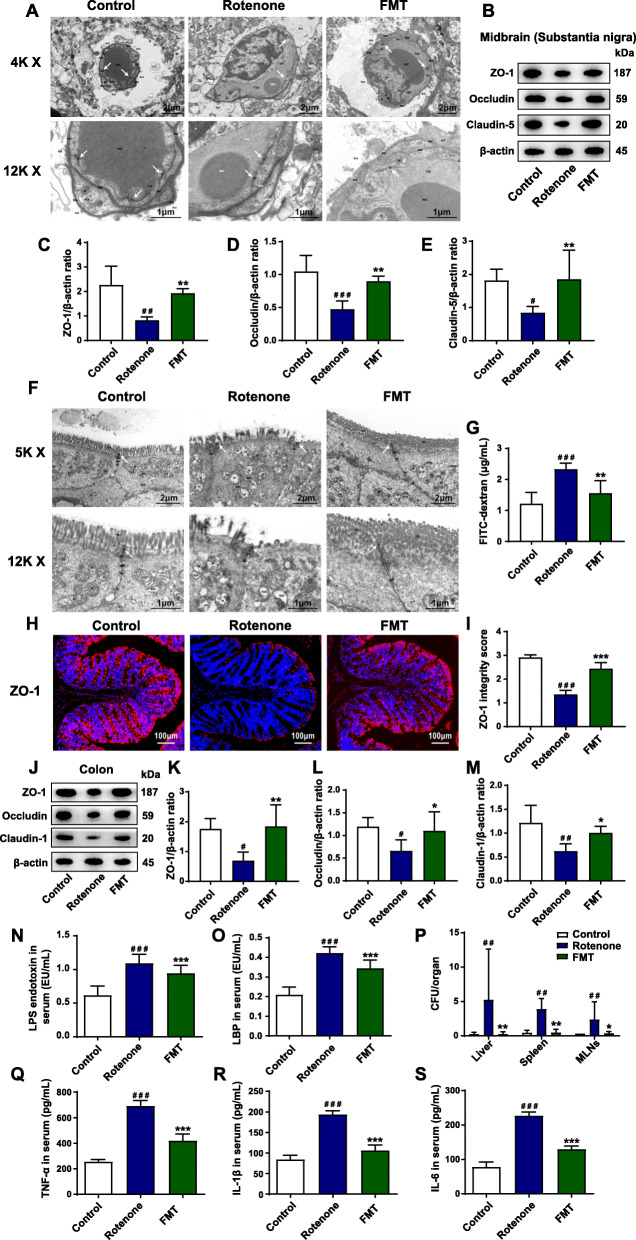
FMT treatment restores BBB and intestinal barrier impairment in the rotenone-challenged mouse model. **A** Representative electron micrographs of the tight junction structures of BBB in the SN. **B** Representative western blot brands of ZO-1, occludin, and claudin-5 in the midbrain containing the SN. **C**–**E** The density analysis of ZO-1, occludin, and claudin-5 western blot in the midbrain containing the SN. **F** Representative electron micrographs of the tight junction structures of intestinal epithelium in the colon. **G** Concentrations of FD4 in serum indicating intestinal permeability. **H** Representative captures of immunofluorescence in the colon of ZO-1. **I** ZO-1 integrity score in the colon. **J** Representative western blot brands of ZO-1, occludin, and claudin-1 in the colon. **K**–**M** The density analysis of ZO-1, occludin, and claudin-1 western blot in the colon. **N** Serum levels of LPS endotoxin. **O** Serum levels of LBP. **P** Numbers of bacteria (CFU) in the livers, spleens, and MLNs of mice. **Q**–**S** Serum levels of inflammatory cytokines, including TNF-α (**Q**), IL-1β (**R**), and IL-6 (**S**). For **C**–**E** and **K**–**M**, *n* = 4 for each group. For **G**, *n* = 5 for each group. For **I**, *n* = 5 for each group. For **N** and **O** and **Q**–**S**, *n* = 8 for each group. For **P**, *n* = 6 for each group. Data are presented as mean ± SD. ^#^*P* < 0.05, ^##^*P* < 0.01, ^###^*P* < 0.001 versus the control group; *P < 0.05, **P < 0.01, ***P < 0.001 versus the rotenone group

It is reported that the destruction of BBB and intestinal barrier is critical in the development of PD [22]. Under normal circumstances, the paracellular gaps are sealed by tight junctions to maintain the integrity and the functions of barriers [53]. To determine whether the neuroinflammation is associated with BBB leakage, we conducted TEM analysis to evaluate the structures of BBB. As demonstrated by the TEM analysis, the tight junctions of BBB were diffuse without organized structures and the endothelial cells were impaired in the rotenone group (Fig. 3A). After the administration of FMT, the structures of tight junctions in the SN were improved to form clear and dense bands and the endothelial cell destruction was also alleviated (Fig. 3A). Further western blot results illustrated that the expression of the 3 major tight junction proteins decreased significantly in the rotenone group, including ZO-1 (*P* < 0.01), occludin (*P* < 0.001), and claudin-5 (*P* < 0.05) compared to the control group whereas FMT treatment markedly restored the reductions (all *P* < 0.01) (Fig. 3B-E). In addition, the immunofluorescence staining results in the SN found that the ZO-1 intensities remarkably reduced in the rotenone group (*P* < 0.001) but markedly elevated in the FMT group (*P* < 0.001) (Fig. S1A-B). Consistently, qPCR analysis on the midbrain samples containing the SN demonstrated the lower mRNA expression of *ZO1* (*P* < 0.01), *Ocln* (*P* < 0.001), and *Cldn5* (*P* < 0.001) in the rotenone group than the control group. By contrast, the expression of these 3 genes (*ZO1*, *P* < 0.01; *Ocln*, *P* < 0.001; *Cldn5*, *P* < 0.001) was significantly increased in the FMT group (Fig. S1C-E).

Besides, the functions and the structures of the intestinal barriers were also tested. First of all, the in vivo intestinal permeability assay was performed, in which the higher intensity of FD4 detected in the serum represents the elevated gut permeability. The increased concentrations of FD4 were shown in the serum of the rotenone group in comparison with the control group (*P* < 0.001) while FMT administration remarkably attenuated the elevation of serum FD4 concentrations (*P* < 0.01) (Fig. 3G). Moreover, we conducted a bacterial translocation experiment to reflect the integrity of the gut barriers. Translocated bacteria were detected in the germ-free organs, including the liver (*P* < 0.01), spleen (*P* < 0.01), and MLNs (*P* < 0.01) of the rotenone group mice while FMT treatment remarkably suppressed the translocation (Liver, *P* < 0.01; Spleen, *P* < 0.01; MLNs, *P* < 0.05) (Fig. 3P). Furthermore, we conducted TEM analysis to detect the structures of intestinal barriers. The results illustrated the impaired epithelium with sparse microvilli, as well as decreased and discontinuous electron-dense materials with wider paracellular gaps in the colon slides of rotenone-challenged mice (Fig. 3F). However, the epithelium was present with regularly organized microvilli and intact tight junctions after FMT treatment (Fig. 3F). Additionally, the expression of tight junction proteins in the colon was also measured by different methods. Immunofluorescence staining results showed disrupted and decreased expression of ZO-1 in the rotenone group (*P* < 0.001) whereas FMT administration significantly preserved the ZO-1 integrity (*P* < 0.001) (Fig. 3H, I). In addition, western blot results demonstrated that the expression of ZO-1 (*P* < 0.05), occludin (*P* < 0.05), and claudin-1 (*P* < 0.01) was remarkably reduced in the colon samples of the rotenone group mice compared to the control group mice but elevated in the FMT group mice (ZO-1, *P* < 0.01; occludin, *P* < 0.05; claudin-1, *P* < 0.05) (Fig. 3J-M). Consistently, mRNA expression of these important genes in the colon also decreased significantly in the rotenone group (all *P* < 0.001) while FMT treatment restored the declined expression (*ZO1*, *P* < 0.01; *Ocln*, *P* < 0.01; *Cldn1*, *P* < 0.001) (Fig. S1F-H).

To further test whether the restoration of the intestinal barriers can lead to the decline of leaked pro-inflammatory molecules, we measured the levels of LPS, LBP, and inflammatory cytokines in the circulation. The ELISA analysis revealed that both serum LPS and LBP in the rotenone group were elevated to more than twice those of the control group (both *P* < 0.001, Fig. 3N-O). By contrast, the levels of LPS (rotenone group, 1.09 EU/mL; FMT group, 0.95 EU/mL) (Fig. 3N) and LBP (rotenone group, 0.42 EU/mL; FMT group, 0.35 EU/mL) (Fig. 3O) in the serum were remarkably reduced by FMT administration (both *P* < 0.001). In addition, significantly higher levels of pro-inflammatory cytokines (TNF-α, IL-1β, and IL-6) were detected in the systemic circulation of the rotenone group compared to the control group (all *P* < 0.001) by ELISA analysis whereas FMT administration restored the elevation (all *P* < 0.001) (Fig. 3Q-S). All these data suggest that FMT treatment preserves the BBB damage and the intestinal barrier impairment in the rotenone-induced mouse model.

### FMT administration inhibits the TLR4/MyD88/NF-κB signaling pathway in the SN and the colon of the rotenone-induced mouse model

**Fig. 4:**
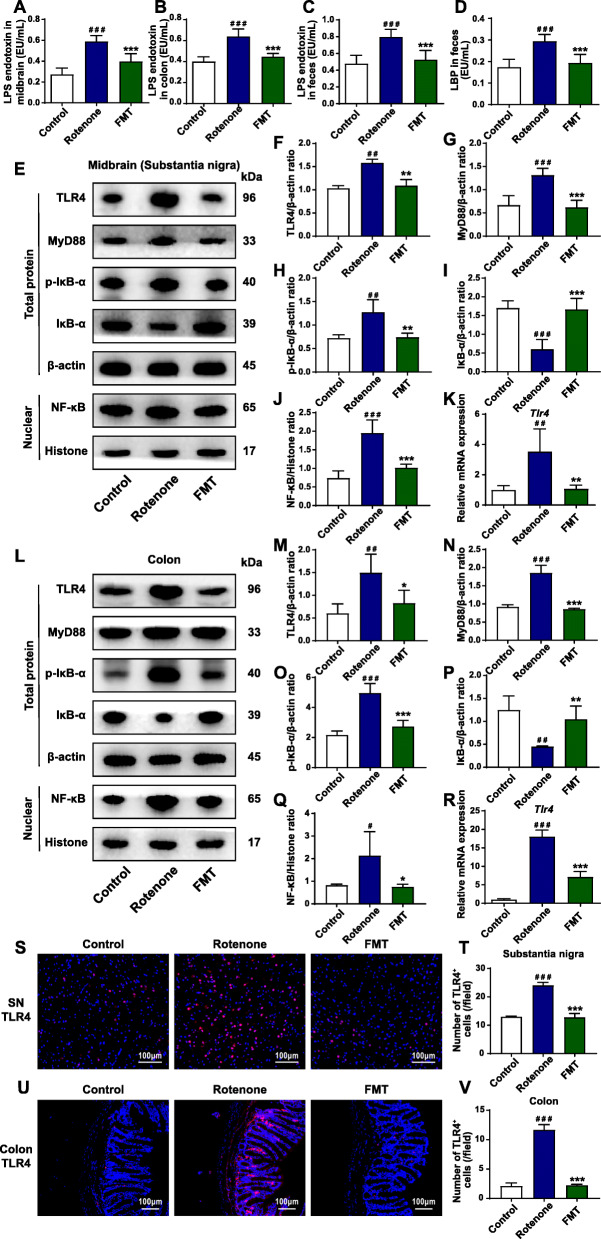
FMT administration inhibits the TLR4/MyD88/NF-κB signaling pathway in the SN and the colon of rotenone-induced PD mouse model. **A** Levels of LPS endotoxin in the midbrain containing the SN. **B** Levels of LPS endotoxin in the colon. **C** Fecal levels of LPS endotoxin. **D** Fecal levels of LBP. **E** Representative western blot brands of TLR4, MyD88, p-IκB-α, IκB-α, and NF-κB in the midbrain containing the SN. **F**–**J** The density analysis results of TLR4, MyD88, p-IκB-α, IκB-α, and NF-κB in the midbrain containing the SN. **K** mRNA expression of *Tlr4* in the midbrain containing the SN. **L** Representative western blot brands of TLR4, MyD88, p-IκB-α, IκB-α, and NF-κB in the colon. **M**–**Q** The density analysis results of TLR4, MyD88, p-IκB-α, IκB-α, and NF-κB in the colon. **R** mRNA expression of *Tlr4* in the colon. **S** Representative captures of immunofluorescence in the SN of TLR4. **T** Numbers of TLR4^+^ cells in the SN. **U** Representative captures of immunofluorescence in the colon of TLR4. **V** Numbers of TLR4^+^ cells in the colon. For **A**–**D**, *n* = 8 for each group. For **F**–**J** and **M**–**Q**, *n* = 4 for each group. For **K** and **R**, *n* = 3 for each group. For **T** and **V**, *n* = 5 for each group. Data are presented as mean ± SD. ^#^*P* < 0.05, ^##^*P* < 0.01, ^###^*P* < 0.001 versus the control group; *P < 0.05, **P < 0.01, ***P < 0.001 versus the rotenone group

We hypothesized that the disrupted barriers can lead to the leakage of pathogenic LPS and LBP, thus activating the TLR4 signaling pathway both in the SN and the colon. To validate the hypothesis, we first detected the levels of LPS in the SN and the colon. In the midbrain tissues containing the SN, the ELISA results demonstrated that LPS elevated more than two times in the rotenone group (*P* < 0.001) compared to the control group but decreased in the FMT group (rotenone group, 0.59 EU/mL; FMT group, 0.40 EU/mL; *P* < 0.001) (Fig. 4A). Consistently in the colon, the LPS levels remarkably increased in the rotenone group (*P* < 0.001) while FMT administration significantly inhibited the levels (rotenone group, 0.64 EU/mL; FMT group, 0.45 EU/mL; *P* < 0.001) (Fig. 4B). What’s more, the rotenone group showed significantly higher levels of LPS and LBP in the fecal pellets (both *P* < 0.001) (Fig. 4C, D). By contrast, the FMT group exerted the remarkably suppressed levels of LPS (rotenone group, 0.80 EU/mL; FMT group, 0.53 EU/mL; *P* < 0.001) (Fig. 4C) and LBP (rotenone group, 0.30 EU/mL; FMT group, 0.19 EU/mL; *P* < 0.001) (Fig. 4D). Capable of recognizing the increased LPS, TLR4 and its downstream pathway have shown the dominance in the mechanisms of the microbiota-gut-brain axis [18, 27]. To examine the activation status of the TLR4 pathway in the SN and the colon, western blot, qPCR, and immunofluorescence staining were performed. In the midbrain containing the SN, the western blot results demonstrated a significantly enhanced expression of TLR4 (*P* < 0.01), MyD88 (*P* < 0.001), p-IκB-α (*P* < 0.01), and NF-κB (*P* < 0.001) and the decreased expression level of IκB-α (*P* < 0.001) in the rotenone group relative to the control group (Fig. 4E-J). Conversely, the FMT group showed the remarkably reduced expression of TLR4 (*P* < 0.01), MyD88 (*P* < 0.001), p-IκB-α (*P* < 0.01), NF-κB (*P* < 0.001) and the increased level of IκB-α expression (*P* < 0.001) (Fig. 4E-J). In addition, the same trends of TLR4 expression were not only confirmed by qPCR (control group vs. rotenone group, *P* < 0.01; rotenone group vs. FMT group, *P* < 0.01) (Fig. 4K) but also by immunofluorescence staining in the SN (control group vs. rotenone group, *P* < 0.001; rotenone group vs. FMT group, *P* < 0.001) (Fig. 4S, T).

Likewise, we measured the TLR4 pathway activation in the colon samples, which was consistent with the results in the midbrain tissues. As revealed by western blot, the rotenone group showed a significantly higher expression of proteins, including TLR4 (*P* < 0.01), MyD88 (*P* < 0.001), p-IκB-α (*P* < 0.001), and NF-κB (*P* < 0.05), as well as the lower expression level of IκB-α protein than the control group (*P* < 0.01) (Fig. 4L-Q). On the contrary, FMT administration illustrated the inhibition of TLR4 pathway in the colon by reducing the expression of TLR4 (*P* < 0.05), MyD88 (*P* < 0.001), p-IκB-α (*P* < 0.001), NF-κB (*P* < 0.05) and increasing the expression level of IκB-α (*P* < 0.01) (Fig. 4L-Q)). Consistently, the relative mRNA expression of *Tlr4* in the colon was also remarkably elevated in the rotenone group (*P* < 0.001) but was suppressed by FMT administration (*P* < 0.001) (Fig. 4R). In addition, the immunofluorescence staining results revealed that the numbers of TLR4^+^ cells notably increased in the rotenone group (*P* < 0.001) whereas FMT treatment significantly reduced the numbers (*P* < 0.001) (Fig. 4U, V). Clearly, all the evidence supports that FMT administration inhibits the TLR4/MyD88/NF-κB signaling pathway activated by LPS both in the SN and the colon of rotenone-challenged mice.

### FMT administration suppresses the generation of pro-inflammatory proteins in the SN and the colon of rotenone-challenged mice

**Fig. 5:**
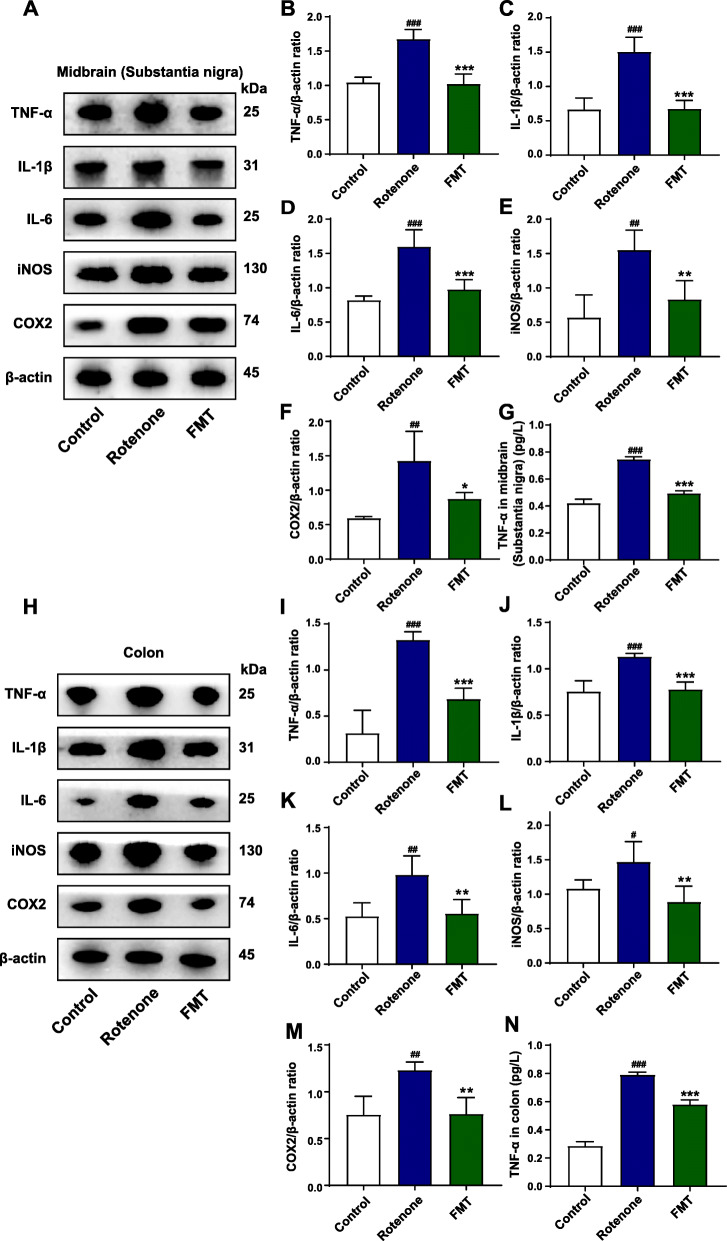
FMT treatment suppresses the generation of pro-inflammatory molecules both in the SN and the colon of rotenone-challenged mice. **A** Representative western blot brands of TNF-α, IL-1β, IL-6, iNOS, and COX2 in the midbrain containing the SN. **B**–**F** The density analysis results of TNF-α, IL-1β, IL-6, iNOS, and COX2 in the midbrain containing the SN. **G** TNF-α levels in the midbrain containing the SN. **H** Representative western blot brands of TNF-α, IL-1β, IL-6, iNOS, and COX2 in the colon. **I**–**M** The density analysis results of TNF-α, IL-1β, IL-6, iNOS, and COX2 in the colon. **N** TNF-α levels in the colon. For **B**–**F** and **I**–**M**, *n* = 4 for each group. For **G** and **N**, *n* = 8 for each group. Data are presented as mean ± SD. ^#^*P* < 0.05, ^##^*P* < 0.01, ^###^*P* < 0.001 versus the control group; *P < 0.05, **P < 0.01, ***P < 0.001 versus the rotenone group

As the downstream transcription factor of TLR4/MyD88 pathway, NF-κB is responsible for stimulating the generation of major pro-inflammatory cytokines (TNF-α, IL-1β, IL-6) and activating the expression of iNOS and COX2 [54]. To further confirm the activation status of TLR4 pathway among different groups, we measured the expression of these 5 representative pro-inflammatory products by different methods. Above all, western blot of the midbrain containing the SN found that the expression of all these 5 proteins (TNF-α, *P* < 0.001; IL-1β, *P* < 0.001; IL-6, *P* < 0.001; iNOS, *P* < 0.01; COX2, *P* < 0.01) were notably enhanced in the rotenone group compared to the control group (Fig. 5A-F). However, the elevated expression was all remarkably inhibited by FMT treatment (TNF-α, *P* < 0.001; IL-1β, *P* < 0.001; IL-6, *P* < 0.001; iNOS, *P* < 0.01; COX2, *P* < 0.05) (Fig. 5A-F). Consistent with the results in the midbrain, the western blot results in the colon also showed that these 5 pro-inflammatory proteins had higher expression (TNF-α, *P* < 0.001; IL-1β, *P* < 0.001; IL-6, *P* < 0.01; iNOS, *P* < 0.05; COX2, *P* < 0.01) in the rotenone group than the control group (Fig. 5H-M), while FMT administration significantly preserved the increased protein expression (TNF-α, *P* < 0.001; IL-1β, *P* < 0.001; IL-6, *P* < 0.01; iNOS, *P* < 0.01; COX2, *P* < 0.01) (Fig. 5H-M). Moreover, the ELISA analysis of TNF-α validated its increased expression in the rotenone group (midbrain containing SN, *P* < 0.001; colon, *P* < 0.001) and its reduced expression in the FMT group (midbrain containing SN, *P* < 0.001; colon, *P* < 0.001) (Fig. 5G and N). Besides, the corresponding mRNA expression of these 5 proteins was also remarkably raised by rotenone administration in the midbrain containing the SN (*Tnf*, *P* < 0.01; *Il1b*, *P* < 0.001; *Il6*, *P* < 0.01; *Nos2*, *P* < 0.01; *COX2*, *P* < 0.01) and the colon (*Tnf*, *P* < 0.01; *Il1b*, *P* < 0.001; *Il6*, *P* < 0.001; *Nos2*, *P* < 0.01; *COX2*, *P* < 0.001) (Fig. S2A-E), which was suppressed by FMT treatment in the midbrain containing the SN (*Tnf*, *P* < 0.01; *Il1b*, *P* < 0.001; *Il6*, *P* < 0.01; *Nos2*, *P* < 0.001; *COX2*, *P* < 0.01) and the colon (*Tnf*, *P* < 0.05; *Il1b*, *P* < 0.01; *Il6*, *P* < 0.001; *Nos2*, *P* < 0.01; *COX2*, *P* < 0.001) (Fig. S2A-E). Collectively, these findings prove that FMT administration suppresses the generation of pro-inflammatory proteins enhanced by TLR4 pathway activation, thus locally inhibiting the inflammation in the SN and the colon.

### FMT treatment alleviates gut microbiota dysbiosis of the rotenone-induced PD mouse model

**Fig. 6:**
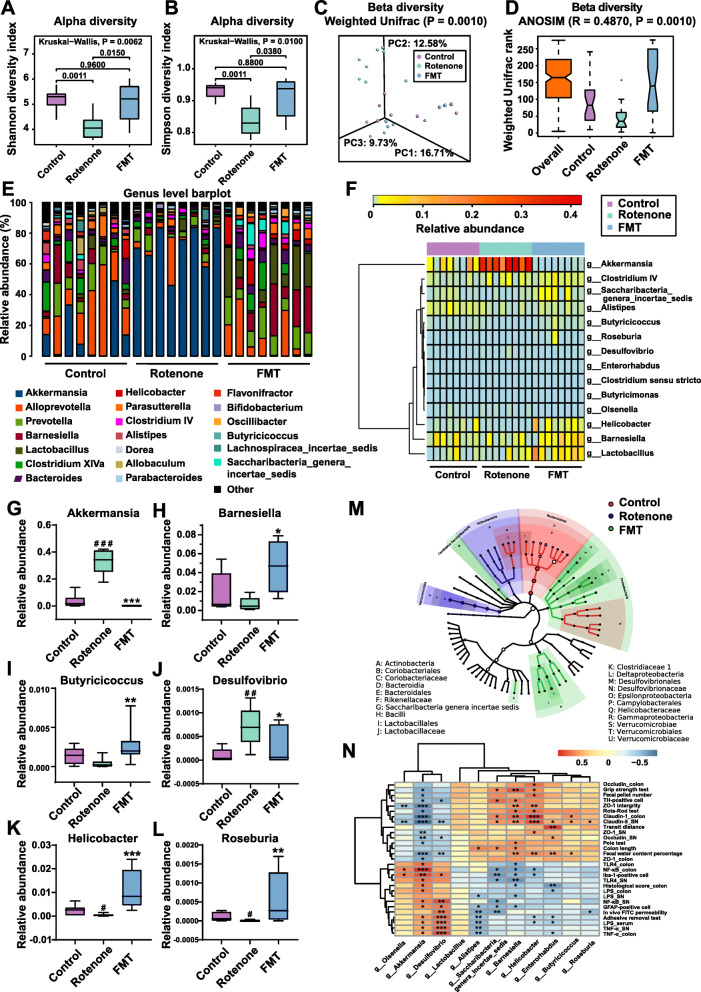
FMT treatment alleviates fecal microbiota dysbiosis of rotenone-induced PD mice. **A** Analysis of alpha diversity of gut microbiota by Shannon analysis. **B** Analysis of alpha diversity of gut microbiota by Simpson analysis. **C** PCoA plots of beta diversity based on weighted UniFrac analysis in different groups. **D** Beta diversity based on weighted UniFrac ANOSIM analysis in different groups. **E** Relative abundances of gut microbiota at the genus level in the 3 groups. **F** Heatmap analysis of relative abundances of gut microbiota at the genus level in different groups. **G**–**L** Relative abundances of 6 significantly altered bacterial genera: *Akkermansia* (**G**), *Barnesiella* (**H**), *Butyricicoccus* (**I**), *Desulfovibrio* (**J**), *Helicobacter* (**K**), *Roseburia* (**L**). **M** Graphical phylogenetic analysis of gut microbiota alterations among 3 groups. The size of each dot represents the relative abundance. **N** Heatmap of the association between microbiota and other experimental results. In this figure, *n* = 8 for each group. Each boxplot represents the median, interquartile range, minimum and maximum values. ^#^*P* < 0.05, ^##^*P* < 0.01, ^###^*P* < 0.001 versus the control group; *P < 0.05, **P < 0.01, ***P < 0.001 versus the rotenone group

Recently, accumulating reports have found gut microbiota dysbiosis in PD animal models and suggest its vital role in the development of PD [20]. To explore how FMT administration protects the rotenone-induced PD mouse model through modulating microbiome community structures, we carried out 16S RNA sequencing on fecal samples of mice from different groups. After 4 weeks of model establishment, the gut microbiota community of the rotenone group was significantly separated from the control group. In addition, remarkable differences in the abundances of some bacterial taxa, such as *Verrucomicrobia* phylum and *Akkermansia* genus, were detected between the 2 groups (data not shown). Then, the feces collected from the mice in different groups at week 6 were analyzed for more detailed information of microbiome alterations. First, the alpha-diversity analysis was conducted to evaluate the richness and diversity of bacterial species. As illustrated in Fig. 6A and B, decreased Shannon index (*P* = 0.0011) and Simpson index (*P* = 0.0011) were present in the rotenone group compared to the control group while the FMT group showed higher indices (Shannon index: *P* = 0.0150; Simpson index: *P* = 0.0380) respectively. Besides, no difference in alpha diversity between the control group and the FMT group was detected (Shannon index: *P* = 0.9600; Simpson index: *P* = 0.8800; Fig. 6A, B). Moreover, beta-diversity based on weighted UniFrac distances was measured to indicate the similarity of microbiota composition in these 3 groups. At week 6, the structures of the rotenone group were significantly separated, which was revealed by the Adonis test (*P* = 0.0010; Fig. 6C) as well as ANOISM analysis (*R* = 0.4870, *P* = 0.0010; Fig. 6D), while the microbiome community structures of the control group and the FMT group were similar.

To further identify the critical bacteria which contributed to the development of PD, we compared the relative abundances of microbes at various taxon levels among different groups. All the detailed data of different microbes at phylum, family and genus levels with relative abundances over 0.01% and overall significances lower than 0.05 were displayed in Table 2. At the phylum level, *Verrucomicrobia* showed a significant increase in the rotenone group compared to the control group (*P* = 0.0002) while FMT treatment markedly reduced the abundance (*P* = 0.0009) (Table 2 & Fig. S3A-B). By contrast, another phylum *Proteobacteria* had lower abundance in the rotenone group than the control group (*P* = 0.0180) but elevated abundance in the FMT group (*P* = 0.0074) (Table 2 & Fig. S3A & C). At the family level, the rotenone group showed the increased abundances of *Verrucomicrobiaceae* (*P* = 0.0002) and *Desulfovibrionaceae* (*P* = 0.0236) as well as a reduction in *Helicobacteraceae* (*P* = 0.0180) and *Rikenellaceae* (*P* = 0.0070) compared to the control group (Table 2 and Fig. S3D-E). In comparison with the rotenone group, the FMT group had the decreased abundances of *Verrucomicrobiaceae* (*P* = 0.0009) and *Coriobacteriaceae* (*P* = 0.0047) as well as the elevated abundances of *Helicobacteraceae* (*P* = 0.0002), *Enterobacteriaceae* (*P* = 0.0349), and *Lactobacillaceae* (*P* = 0.0070) (Table 2 and Fig. S3D-F).

**Table 2:** Relative abundance of microbiota at different taxon levels

Taxonomic level	Relative abundance			*P* value			
	Control	Rotenone	FMT	Overall	Con vs. Rot	Rot vs. FMT	Con vs. FMT
Phylum							
*Bacteroidetes*	0.6148	0.4189	0.5962	0.0455	0.0104*	0.1049	0.8785
*Candidatus Saccharibacteria*	0.0032	0.0014	0.0125	0.0191	0.1304	0.0104*	0.0927
*Proteobacteria*	0.0266	0.0079	0.0314	0.0105	0.0180*	0.0074**	0.7984
*Verrucomicrobia*	0.0343	0.3284	0.0008	0.0001	0.0002***	0.0009***	0.0404*
Family							
*Coriobacteriaceae*	0.0025	0.0024	0.0010	0.0109	1.0000	0.0047**	0.0574
*Desulfovibrionaceae*	0.0004	0.0014	0.0015	0.0142	0.0236*	0.6358	0.0653
*Enterobacteriaceae*	0.0034	0.0001	0.0005	0.0485	0.0574	0.0349*	0.3717
*Helicobacteraceae*	0.0054	0.0005	0.0223	0.0008	0.0180*	0.0002***	0.1949
*Lactobacillaceae*	0.0073	0.0096	0.0583	0.0054	0.5054	0.0070**	0.0574
*Rikenellaceae*	0.0087	0.0038	0.0046	0.0124	0.0070**	0.4005	0.4005
*Verrucomicrobiaceae*	0.0343	0.3284	0.0008	0.0001	0.0002***	0.0009***	0.0404*
Genus							
*Akkermansia*	0.0343	0.3284	0.0008	0.0001	0.0002***	0.0009***	0.0404*
*Alistipes*	0.0087	0.0037	0.0045	0.0095	0.0086**	0.4005	0.1304
*Barnesiella*	0.0182	0.0070	0.0372	0.0427	0.1949	0.0207*	0.1605
*Butyricicoccus*	0.0014	0.0004	0.0027	0.0108	0.0653	0.0053**	0.2069
*Desulfovibrio*	0.0001	0.0010	0.0003	0.0066	0.0018**	0.0500*	0.5369
*Helicobacter*	0.0054	0.0005	0.0223	0.0008	0.0180*	0.0002***	0.1949
*Lactobacillus*	0.0073	0.0096	0.0583	0.0054	0.5054	0.0070**	0.0574
*Saccharibacteria genera incertae sedis*	0.0032	0.0014	0.0125	0.0191	0.1304	0.0104*	0.0927

*P < 0.05, **P < 0.01, ***P < 0.001. Statistical analysis by the Kruskal-Wallis multiple comparison test with the post-hoc Mann-Whitney *U* test

As demonstrated in Fig. 6E and F and Table 2, the overall significant differences among the 3 groups were detected in the genera *Akkermansia* (*P* = 0.0001), *Alistipes* (*P* = 0.0095), *Barnesiella* (*P* = 0.0427), *Butyricicoccus* (*P* = 0.0108), *Desulfovibrio* (*P* = 0.0066), *Helicobacter* (*P* = 0.0008), *Lactobacillus* (*P* = 0.0054), and *Saccharibacteria genera incertae sedis* (*P* = 0.0191). We further selected 6 genera and utilized boxplots to display their detailed information (Fig. 6G-L). Compared to the control group, the genera *Akkermansia* (*P* = 0.0002; Fig. 6G) and *Desulfovibrio* (*P* = 0.0018; Fig. 6J) showed significantly elevated relative abundances whereas the genera *Helicobacter* (*P* = 0.0180; Fig. 6K) and *Roseburia* (*P* = 0.0124; Fig. 6L) had the notably decreased abundances in the rotenone group. However, FMT treatment remarkably reduced the abundances of *Akkermansia* (*P* = 0.0009; Fig. 6G) and *Desulfovibrio* (*P* = 0.0500; Fig. 6J), while the increased relative abundances of *Barnesiella* (*P* = 0.0207; Fig. 6H), *Butyricicoccus* (*P* = 0.0053; Fig. 6I), *Helicobacter* (*P* = 0.0002; Fig. 6K) and *Roseburia* (*P* = 0.0029; Fig. 6L) were witnessed in the FMT group. In addition, the comparisons of bacteria at different taxon levels between the control group and the FMT group revealed that most of them had no significant difference, suggesting that these 2 groups shared similar gut microbiome profiles (Table 2 and Fig. 6E-L and Fig. S3A-F). Collectively, differential abundances of gut microbes at various taxon levels among all the 3 groups are summarized in Fig. 6M.

To better interpret the functions of these significantly altered bacteria, we performed a functional analysis of KEGG pathway enrichment. A total of 21 KEGG pathways at level 2 were identified (Fig. S3G). Among them, the immune system was enriched in the control group. Meanwhile, pathways such as cellular processes and signaling, xenobiotics biodegradation and metabolism, and lipid metabolism were enriched in the rotenone group. Besides, replication and repair, nucleotide metabolism, translation, and other pathways were enriched in the FMT group (Fig. S3G). Additionally, level 3 analysis revealed that 2 pathways (DNA repair and recombination proteins and energy metabolism) were enriched in the control group whereas 17 pathways, including bacterial secretion system, secretion system, lipopolysaccharide biosynthesis, transcription machinery, replication recombination, and repair proteins, were enriched in the rotenone group (Fig. S3H). In addition, there were 16 level 3 pathways enriched in the FMT group, including pyrimidine metabolism, ribosome, methane metabolism, and chromosome (Fig. S3H). The correlations between the abundances of bacterial genera and other experimental results were analyzed to further demonstrate the effects of microbiota on the rotenone-challenged mice. As illustrated in Fig. 6N, the abundances of some genera like *Akkermansia*, *Barnesiella*, *Desulfovibrio*, and *Helicobacter* showed significant correlations with many other experimental results, including the behavioral tests, the GI function tests, the TH-positive cell numbers in the SN, and some inflammation-related data. Together, these data provide evidence that FMT administration attenuates microbiota dysbiosis to protect the rotenone-intoxicated mice, suggesting the critical role of microbiota dysbiosis in the development of PD.

### Correlations support the involvement of the microbiota-gut-brain axis in the rotenone-challenged mouse model

**Fig. 7:**
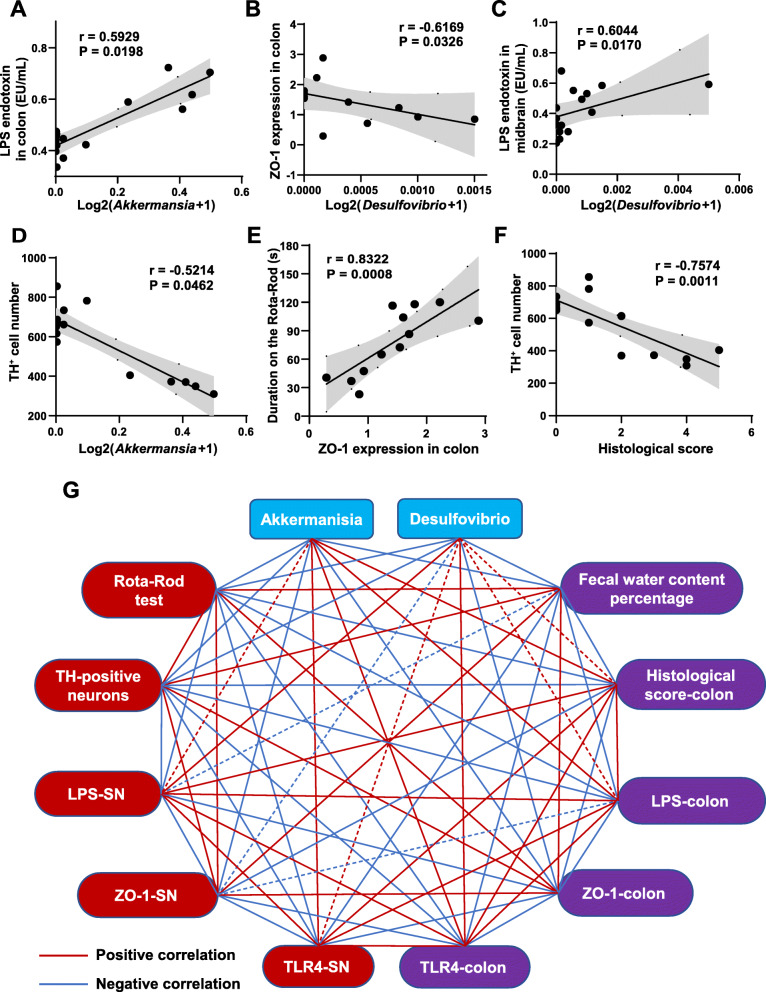
Correlations among different results support the possible involvement of the microbiota-gut-brain axis in the development of the rotenone-induced mouse model. **A** LPS levels in the colon were positively correlated with relative abundance of *Akkermansia.* **B** Relative expression of ZO-1 in the colon detected by western blot was inversely correlated with relative abundance of *Desulfovibrio.* **C** Levels of LPS in the midbrain containing the SN were significantly positively correlated with relative abundance of *Desulfovibrio*. **D** TH^+^ cell numbers were negatively correlated with relative abundance of *Akkermansia.* **E** Rota-Rod test data were significantly positively correlated with relative expression of ZO-1 in the colon detected by western blot. **F** TH^+^ cell numbers were negatively correlated with H&E histological scores in the colon. **G** Correlations among different experimental results of microbiota-gut-brain axis. The blue nodes represent gut microbiota. The red nodes represent the results in the SN of the mouse model and the purple nodes represent the data in the colon. The red lines represent positive correlations while the blue lines represent negative correlations. The solid lines mean significant correlations, and the dotted lines represent no correlations

To better understand the mechanisms of the microbiota-gut-brain axis in the pathogenesis of the rotenone-induced PD model and the protective effects of FMT treatment, we performed correlation analysis between several experimental results. The results demonstrated that the levels of LPS in the colon were significantly positively correlated with the relative abundance of *Akkermansia* (*r* = 0.5929, *P* = 0.0198) (Fig. 7A). Besides, the relative abundance of *Desulfovibrio* was inversely correlated with the ZO-1 expression in the colon (*r* = − 0.6169, *P* = 0.0326) (Fig. 7B) and was positively correlated with LPS levels in the midbrain containing the SN (*r* = 0.6044, *P* = 0.0170) (Fig. 7C). We also witnessed a significantly negative correlation between the relative abundance of *Akkermansia* and TH^+^ cell numbers in the SN region (*r* = − 0.5214, *P* = 0.0462) (Fig. 7D). In addition, the ZO-1 expression in the colon was found to be remarkably positively associated with the performances of the Rota-Rod test (*r* = 0.8322, *P* = 0.0008) (Fig. 7E). Also, the negative correlation between the TH^+^ cell numbers in the SN and the H&E histological scores of the colon was observed in our study (*r* = − 0.7574, *P* = 0.0011) (Fig. 7F). The correlations between more results of interest were conducted and displayed as a network in Fig. 7G. The relative abundances of *Akkermansia* and *Desulfovibrio* represented microbiota changes in different groups. The duration on the rod, the TH^+^ neuron numbers in the SN region, the levels of LPS, the expression of ZO-1 and TLR4 in the midbrain containing the SN were chosen to represent the alterations in the brain. Additionally, we selected the fecal water content percentages, the H&E histological scores, the levels of LPS, as well as the expression of ZO-1 and TLR4 in the colon as the representative features of the gut. Interestingly, we found that most correlations between these markers were statistically significant. These correlation results collectively support the involvement of the microbiota-gut-brain axis in the development of the chronic rotenone-challenged mouse model and the protective effects of FMT treatment on this model.

## Discussion

Given that gut microbiota dysbiosis is increasingly reported to play a significant role in PD pathogenesis [13, 55], we established a PD mouse model associated with microbiota dysbiosis by the oral administration of rotenone to evaluate the protective effects of FMT treatment on PD and to further explore the possible mechanisms. Targeting at mitochondrial complex I, rotenone has been widely utilized to induce PD models [56]. One study revealed the decline of the fecal pellet output and the TH-positive neurons in the mice orally administrated with rotenone [32]. Consistent with a longitudinal study using the same model [38], our previous study found that this chronic rotenone-induced mouse model showed GI dysfunctions prior to the motor deficits, which suggested that PD might stem from the gut. As illustrated in our current research, the rotenone-challenged mice had significant weight loss, GI dysfunctions (indicated by the decreased intestinal transit distance, the lower fecal water content, the declined fecal pellet output frequency, and the reduced colon length), and motor deficits (indicated by the poorer performances in Rota-Rod tests, pole tests, adhesive removal tests, and grip strength tests) compared to the control group. H&E analysis of the colon tissues suggested that the GI dysfunctions may result from immune infiltration and epithelium damage caused by rotenone in the gut. Further histological analysis of the brain tissues revealed that rotenone intoxication caused the degeneration of TH-positive cells (dopaminergic neurons) and the cytoplasmic accumulation of α-syn in the SN region, which is consistent with other studies [33–36]. Taken together, it is suggested that the chronic rotenone-induced mouse model can well mimic the progression of PD, present with both GI dysfunctions and motor deficits.

Furthermore, several investigations demonstrated abnormal microbial community structures in the rotenone-challenged PD mice [37, 38, 57, 58]. Besides, the altered gut microbial community is also present in PD patients, like increased *Akkermansia* genus, *Firmicutes* phylum, and decreased *Prevotella copri*, *Prevotellaceae* [7, 55]. Strikingly, a recent study revealed that oral administration of rotenone resulted in gut microbiota dysbiosis, disrupted intestinal barrier, and PD-like motor impairments in conventionally raised mice but not in germ-free mice, highlighting the significant role of gut microbiota dysbiosis in PD [39]. Here, in our study, we detected the gut microbiota composition of the PD mice by 16S RNA sequencing analysis. At week 4, the alterations in gut microbiota structure began to occur in the rotenone-induced mice in consistency with the GI dysfunctions present at that time. Later at week 6, alpha diversity decreased in the rotenone group, which is also witnessed in some other PD models [38, 59]. However, some studies showed no changes in alpha diversity between PD patients and healthy controls [14, 60], which may be caused by several reasons. Since alpha diversity may change dynamically during the development of PD [61], microbiota detection may generate different results at different stages of PD. Additionally, beta diversity results also suggested a remarkable change in the microbiota composition of rotenone-induced mice. At various taxon levels, the rotenone-challenged mice showed the increased *Verrucomicrobia* phylum and *Verrucomicrobiaceae* family as well as the decreased *Bacteroidetes* phylum, which were also witnessed in PD patients [13, 14, 62, 63]. Besides, the *Akkermansia* genus, belonging to the *Verrucomicrobia* phylum and *Verrucomicrobiaceae* family, showed a significant increase in the rotenone group. The same change was detected not only in a study of the rotenone-challenged PD mouse model [37] but also in the fecal samples of PD patients [13, 62, 64, 65]. These data suggested the close association between *Akkermansia* and PD development. Intriguingly, the significant role of *Akkermansia* was also witnessed in another neurological disorder, MS. Although the elevated *Akkermansia* was found in MS patients, the isolated *Akkermansia* strains from patients or its expansion resulting from microRNAs both exerted protective effects on the MS animal model [66, 67]. These findings provide novel perspectives for deeper research of the role of the *Akkermansia* genus in PD pathogenesis. What’s more, some other bacterial genera changed in our study have also been reported to be associated with PD, including *Alistipes* [55], *Barnesiella* [60], *Butyricicoccus* [68], and *Roseburia* [13, 62]. Their associations with gut inflammation may contribute to the alterations in PD [69–72]. Moreover, our study detected reduced *Helicobacter* in the rotenone-challenged mice. To the best of our knowledge, it is the first time to reveal the alteration in the *Helicobacter* genus abundance by 16S RNA sequencing in PD animal models. Although some reports found higher prevalence of Hp infection in PD patients [73–75], they only revealed the correlation between them and no causal relationship has been found for now. In addition, the variability in microbiota community structures among different studies may be a result of several reasons including different hosts, microbiota sampling sites, and sample sequencing. All these factors may lead to different results between patients and animal models. To be noted, the genus *Desulfovibrio* is for the first time found to be relevant to PD by our research. Together, the specific mechanisms of these bacterial taxa in the development of PD still need more investigations.

To test our hypothesis that gut microbiota dysbiosis is necessary in PD genesis, a preliminary study in our laboratory monitored the effects of FMT administration from rotenone-induced PD mice to healthy mice, showing that the mice receiving gut microbiota from PD mice exerted both GI dysfunctions and motor deficits. In addition, dopaminergic neuronal death was detected in the SN of recipients. Moreover, LPS levels were found to elevate in the feces and serum after FMT treatment, suggesting the importance of LPS in the dialogue between the gut and brain. Consistent with the above, some published data also revealed that FMT treatment from PD diseased donors to healthy recipients could cause PD symptoms [16–18], supporting the significant role of gut microbiota dysbiosis in PD pathogenesis.

However, no therapies targeting the microbiota dysbiosis have been approved by FDA to treat PD patients clinically so far. FMT, a method which introduces gut microbiota from a healthy individual to a patient, can re-establish a stable gut microbial community to treat intractable GI diseases, like inflammatory bowel disease and CDI [28]. Since accumulating evidence has supported the close interaction between the gut microbiota and the brain [23, 76], FMT administration has also been applied to some neurodegenerative disorders. For example, a recent investigation demonstrated that FMT administration could protect an Alzheimer’s disease (AD) mouse model [77]. Also, FMT treatment showed neuroprotective effects on a PD patient [31]. However, the protective effects of FMT administration on PD and the possible mechanisms are scarcely investigated. Based on the significant role of microbiota dysbiosis in PD genesis, our study for the first time utilized the chronic oral rotenone-challenged PD mouse model, an appropriate model closely related to gut flora dysbiosis, to evaluate the protective effects of FMT treatment on PD and to elucidate the underlying mechanisms. In our study, FMT treatment demonstrated remarkable effects on alleviating the body weight loss, GI dysfunctions, and motor deficits of the rotenone-induced mice. Besides, FMT administration significantly attenuated the inflammation infiltration and epithelium damage in the gut as well as the pathological hallmarks of the PD mice. Collectively, our present research for the first time proved that FMT treatment protects the rotenone-induced PD mouse model, indicating its potential role as an effective treatment for PD.

Further microbiota analysis suggested that the protective effects of FMT treatment might be mediated by reconstructing the normal gut microbiota. From the general aspect, the alpha-diversity and beta-diversity results showed that the microbial community of the FMT group was similar to the control group. Besides, the comparisons at various taxon levels between the control group and the FMT group revealed no significant differences in gut microbiota profiles between the healthy donors and the recipients, suggesting that FMT administration restored the healthy microbiota in the rotenone-induced PD mice. Moreover, FMT treatment could remarkably change the abundances of some important inflammation-related microbiota taxa in rotenone-induced PD mice. Notably, the *Akkermansia* abundance was elevated in the rotenone group and remarkably decreased after FMT administration, indicating its potential role in the protective effects of FMT treatment on PD. According to other publications, *Akkermansia* is a gram-negative bacterium which can increase the susceptibility of the gut to pathogens by degrading the intestinal mucus barrier to cause local inflammation [78, 79]. Besides, the *Akkermansia* abundance is reported to be negatively correlated with the NLRP6 expression, an innate immune receptor which alleviates intestinal impairments [79]. All these findings suggest that the increased *Akkermansia* is closely associated with intestinal inflammation and further PD symptoms. More in-depth investigations are needed to test and verify whether this bacterium is a potential bacterial target of FMT treatment in PD. In addition, although some studies reported the increase of *Lactobacillus* genus in PD patients [12, 13], controversial results were observed in some other research [65, 68, 80]. Intriguingly, we found that there was no significant difference in the *Lactobacillus* genus after rotenone treatment, and its abundance was elevated after FMT administration. Based on current reports, it is still uncertain whether the increase of *Lactobacillus* is responsible for the initiation and development of PD. As a well-known probiotic genus with anti-inflammatory effects [81], *Lactobacillus* was reported to increase after FMT treatment in colitis models [82, 83], which is consistent with our findings. Further studies need to determine the alterations in the *Lactobacillus* genus after FMT treatment in PD. Collectively, our data support that FMT administration can protect the rotenone-challenged mice by re-establishing a normal microbial community.

To further explore the specific microbiota-related mechanisms in the protective effects of FMT treatment, we conducted multiple measurements regarding the interaction between the gut and the brain. We focused our research on how inflammation mediated the communication between these two distant organs since inflammation plays a critical role in the microbiota-gut-brain axis [84]. As demonstrated by KEGG analysis, some pro-inflammatory pathways, such as secretion system, LPS biosynthesis, xenobiotics biodegradation, and metabolism, were enriched in the PD mice, which is consistent with other studies in PD animal models or patients [13, 38, 62]. This suggested that the dysbiosis of inflammation-related microbes induced by rotenone treatment may lead to chronic inflammation by elevating LPS levels in the gut. In consistence with KEGG results, our ELISA analysis confirmed that fecal and colonic LPS levels remarkably increased in the rotenone group. Thereafter, the elevated LPS can be recognized by TLR4 and then stimulates the following MyD88-dependent NF-κB signaling pathway in the colon of the rotenone group, which was validated by immunofluorescence staining, western blot, and qPCR. The importance of the TLR4 pathway in the microbiota-gut-brain axis has been proven by several previous studies [18, 27]. As the downstream products of NF-κB, the generation of pro-inflammatory cytokines (e.g. TNF-α, IL-1β, and IL-6) [85–87] as well as inflammatory enzymes including iNOS [88] and COX2 [89] can be elevated by the activated TLR4 signaling pathway, which was also detected in the colon of rotenone-challenged mice in our study. On the contrary, FMT treatment remarkably reduced the LPS levels, thus suppressing the TLR4/MyD88/NF-κB signaling pathway and subsequent generation of the pro-inflammatory factors in the colon. Collectively, our study suggests that the suppression of intestinal inflammation induced by bacterial elements through the LPS-activated TLR4/MyD88/NF-κB signaling pathway in the gut may play a critical role in the protective effects of FMT administration on PD.

Recently, a report further suggested that the increased gut permeability which may cause the motor deficits in the rotenone-intoxicated mice is dependent on the gut microbiota [39]. Consistently, the results of the bacterial translocation study and the in vivo intestinal permeability assay in our study found that the intestinal inflammation derived from gut microbiota dysbiosis resulted in disrupted colonic epithelial permeability, which could be alleviated by FMT treatment. Besides, the ELISA analysis of LPS in the colon and the serum validated the above conclusion. More importantly, the TEM analysis and the expression detection of major tight junction proteins by various methods suggested that FMT administration protected the intestinal barrier mainly by restoring the function of tight junctions. All these data collectively verify that FMT treatment can preserve the impaired intestinal barriers and the increased gut permeability in the rotenone-induced mice.

It is increasingly evident that the systemic inflammation caused by the gut inflammation and intestinal barrier damage is vital for the communication between the gut and the brain [23, 90]. In the current study, the translocated bacteria detected in the germ-free organs of rotenone-induced mice indicated that pathogenic microbes were leaked into the systemic circulation. Moreover, our ELISA analyses of LPS, LBP, and 3 important cytokines (TNF-α, IL-1β, and IL-6) in the serum verified that the pro-inflammatory factors leaked into the circulation could induce the systemic inflammation. Accordingly, our findings suggested that the sustained intestinal barrier impairments associated with the gut microbiota dysbiosis could leak microbes, LPS, and pro-inflammatory cytokines into the circulation, thus leading to systemic inflammation. However, FMT treatment inhibited the leakage of the inflammatory molecules, thus suppressing the systemic inflammation.

As reported by other studies, the activated systemic inflammation can interfere with the BBB to make the pro-inflammatory molecules (including cytokines and LPS) get access to the central nerve system (CNS) [91, 92]. In the present study, the LPS levels increased in the SN of the rotenone-challenged mice but decreased after FMT administration, indicating that FMT treatment might reduce the systemic inflammation to restore the BBB integrity damaged by rotenone intoxication, which was further confirmed by the TEM analysis and the expression of 3 major tight junction proteins detected in the SN tissues. As a consequence of impaired BBB in the rotenone-challenged mice, LPS reached the SN region and locally stimulated the TLR4/MyD88/NF-κB pathway, thus enhancing the expression of pro-inflammatory cytokines (TNF-α, IL-1β, and IL-6) and enzymes (iNOS and COX2). However, we observed that FMT treatment inhibited the TLR4 signaling pathway and its downstream product generation in the SN. Stimulated by the TLR4 signaling pathway, the overactivated microglial cells and astrocytes are important for neuroinflammation and dopaminergic neurodegeneration in PD pathogenesis [51, 52, 93]. The immunofluorescence staining results suggested that the activation of microglia and astrocytes correlates with the decrease of TH-positive neurons in the SN. On the contrary, FMT treatment suppressed the overactivated microglia and astrocytes to protect the dopaminergic neurons. Taken together, FMT treatment can inhibit the systemic inflammation, restore the BBB impairment, and suppress the neuroinflammation in the rotenone-induced mice, thus attenuating the neurodegeneration.

**Fig. 8:**
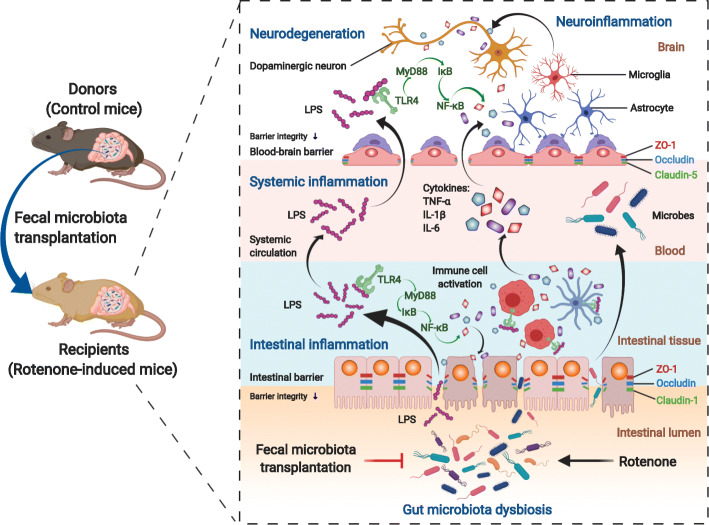
Schematic diagram of the protective effects of FMT administration on the rotenone-induced PD mouse model through the microbiota-gut-brain axis. Briefly, FMT treatment restores the gut microbiota dysbiosis induced by rotenone, which decreases the generation of pathogenic LPS in the gut and inhibits the intestinal inflammation by downregulating the TLR4 signaling pathway. Then, the reduced intestinal barrier permeability decreases the leakage of microbes, LPS, and pro-inflammatory cytokines (e.g., TNF-α, IL-1β, and IL-6) into the systemic circulation. As a consequence of reduced systemic inflammation, lower levels of LPS and pro-inflammatory cytokines get access to the SN across the BBB, which inhibits neuroinflammation (activated astrocytes and microglia) through suppressing LPS-TLR4 signaling pathway activation in the SN. Eventually, the dopaminergic neuronal death is attenuated in the SN. This diagram demonstrates that FMT treatment can correct the gut microbiota dysbiosis and ameliorate the rotenone-induced PD mouse model, in which suppression of the inflammation mediated by the LPS-TLR4 signaling pathway both in the gut and the brain possibly plays a significant role. Further, it is shown that microbiota dysbiosis is involved in the genesis of PD via the microbiota-gut-brain axis

To further validate the importance of the microbiota-gut-brain axis in the current study, we performed correlation analysis between different representative results. The strong associations indicate that microbial community structures influence the close interaction between the gut and the brain. As shown in Fig. 8, the PD-related gut microbiota dysbiosis induced by rotenone causes the LPS-mediated intestinal inflammation and disrupts the intestinal barrier function through activating TLR4/MyD88/NF-κB signaling pathway. The sustained intestinal barrier destruction which can leak microbes, LPS, and pro-inflammatory cytokines (IL-1β, IL-6, and TNF-α) into the circulation leads to systemic inflammation. As a consequence of activated systemic inflammation, higher levels of pro-inflammatory cytokines and LPS get into the SN across BBB, enhancing the activation of microglia and astrocytes (increased Iba-1^+^ and GFAP^+^ cells) through the TLR4/MyD88/NF-κB signaling pathway. Hence, increased pro-inflammatory cytokines are generated in the SN, locally resulting in neuroinflammation. Eventually, neurodegeneration characterized by dopaminergic neuronal death occurs in the SN. On the contrary, FMT administration restores the gut microbiota community and arrests the following effects in the rotenone-challenged mice, finally protecting the PD mouse model. Collectively, we elucidate that FMT treatment attenuates the gut microbiota dysbiosis and protects the PD model, in which suppression of the inflammation mediated by the LPS-TLR4 signaling pathway both in the gut and the brain possibly plays a significant role. Further, we prove that rotenone-induced microbiota dysbiosis is involved in the genesis of PD via the microbiota-gut-brain axis.

By investigating the underlying mechanisms of protective effects of FMT treatment on PD, this study also partially explains the mechanisms of the microbiota-gut-brain axis in the progression of PD. Although it is increasingly evident that the intestinal inflammation and barrier alterations are associated with neurodegeneration in multiple neurological disorders, including PD [39, 94], AD [95, 96], MS [97], and amyotrophic lateral sclerosis [98], specific pathways and mechanisms involved in the interaction between the gut and the CNS still remain unclear. Based on our current results, we speculate that the intestinal inflammation induced by bacterial elements through the LPS-activated TLR4/MyD88/NF-κB signaling pathway together with the following sustained and relatively severe disruption of the intestinal barrier that can leak pro-inflammatory factors into the circulation may contribute to neuroinflammation, ultimately leading to neurodegeneration in the CNS. Further in-depth studies are necessary to test and verify our findings. Besides, our current data on immune cells mainly indirectly reveal their activation status. More illustrations of immune cell data are needed to help us build stronger evidence to prove our conclusions. Therefore, the relevant investigation will be conducted in our future studies.

## Conclusions

Our study for the first time reveals the protective effects of FMT treatment on a chronic rotenone-induced PD mouse model. Further mechanistic studies demonstrate that FMT administration reverses the gut microbiota dysbiosis and protects the PD mouse model, in which suppression of the inflammation mediated by the LPS-TLR4 signaling pathway both in the gut and the brain possibly plays a significant role. Moreover, we prove the significance of microbiota dysbiosis in rotenone-induced PD pathogenesis and supplement the underlying mechanisms of the microbiota-gut-brain axis in the development of PD.

## Supplementary Information

**Additional file 1: Fig. S1** FMT treatment alleviates the expression of tight junction proteins reduced in the rotenone-challenged mouse model. (A) Representative captures of immunofluorescence of ZO-1 in the SN. (B) The intensity analysis of ZO-1 immunofluorescence staining in the SN. (C-E) mRNA expression of tight junction proteins *ZO1*, *Ocln* and *Cldn5* in the midbrain containing the SN*.* (F-H) mRNA expression of tight junction proteins *ZO1*, *Ocln* and *Cldn1* in the colon*.* For (B), *n =* 5 for each group. For (C-H), *n =* 3 for each group. Data are presented as mean ± SD. ## *p <* 0.01, ### *p <* 0.001 versus the control group; ** *p <* 0.01, *** *p <* 0.001 versus the rotenone group.

**Additional file 2: Fig. S2** FMT administration suppresses the generation of pro-inflammatory molecules both in the SN and the colon of rotenone-challenged mice. (A-E) mRNA expression of pro-inflammatory cytokines (*Tnf*, *Il1b, Il6, Nos2, COX2*) in the midbrain containing the SN and the colon. For (A-E), *n =* 3 for each group. Data are presented as mean ± SD. ## *p <* 0.01, ### *p <* 0.001 versus the control group; * *p <* 0.05, ** *p <* 0.01, *** *p <* 0.001 versus the rotenone group.

**Additional file 3: Fig. S3** FMT treatment attenuates microbiota dysbiosis of rotenone-intoxicated mouse model. (A) Relative abundances of gut microbiota at the phylum level in the 3 groups. (B-C) Relative abundances of significantly altered bacterial phyla: *Verrucomicrobia* and *Proteobacteria*. (D) Relative abundances of gut microbiota at the family level in the 3 groups. (E-F) Relative abundances of significantly altered bacterial families: *Verrucomicrobiaceae* and *Lactobacillaceae*. (G) Bar graph of LDA scores of enriched KEGG pathways at level 2. LDA scores (log10) > 2 and *P <* 0.05 are shown. (H) Bar graph of LDA scores of enriched KEGG pathways at level 3. LDA scores (log10) > 2.7 and *P <* 0.05 are shown. In this figure, *n =* 8 for each group. Each boxplot represents the median, interquartile range, minimum and maximum values. # *p <* 0.05, ### *p <* 0.001 versus the control group; ** *p <* 0.01, *** *p <* 0.001 versus the rotenone group.

## Data Availability

The 16S RNA gene sequencing data in this study are available in the Sequence Read Archive (SRA) under project number PRJNA682813.

## Abbreviations

AD: Alzheimer’s disease
ANOSIM: Adonis and analysis of similarity
ASD: Autism spectrum disorder
α-syn: α-Synuclein
BBB: Blood-brain barrier
CDI: *Clostridioides difficile* infection
CFU: Colony-forming units
CMC: Carboxymethylcellulose
COX2: Cyclooxygenase 2
ELISA: Enzyme-linked immunosorbent assay
FD4: FITC-dextran (MW: 4 kDa)
FITC: Fluorescein isothiocyanate
FMT: Fecal microbiota transplantation
GFAP: Glial fibrillary acidic protein
GI: Gastrointestinal
H&E: Hematoxylin and eosin
Hp: *Helicobacter pylori*
Iba-1: Ionized calcium-binding adapter molecule 1
IL-1β: Interleukin-1β
IL-6: Interleukin-6
KEGG: Kyoto Encyclopedia of Genes and Genomes
LBP: Lipopolysaccharide-binding protein
LDA: Linear discriminant analysis
LPS: Lipopolysaccharide
MLNs: Mesenteric lymph nodes
MS: Multiple sclerosis
OTU: Operational taxonomic unit
PCoA: Principal coordinate analysis
PD: Parkinson’s disease
PICRUSt: Phylogenetic Investigation of Communities by Reconstruction of Unobserved States
qPCR: Quantitative polymerase chain reaction assay
SD: Standard deviation
SDS-PAGE: Sodium dodecyl sulfate polyacrylamide gel electrophoresis
SN: Substantia nigra
TEM: Transmission electron microscopy
TH: Tyrosine hydroxylase
TLR: Toll-like receptor
TNF-α: Tumor necrosis factor-α
