# Supplementary figures and images for: Fecal microbiota transplantation protects rotenone-induced Parkinson’s disease mice via suppressing inflammation mediated by the lipopolysaccharide-TLR4 signaling pathway through the microbiota-gut-brain axis
Source: Microbiome. 2021 Nov 17;9:226. doi: 10.1186/s40168-021-01107-9 (PMC8597301; doi:10.1186/s40168-021-01107-9)

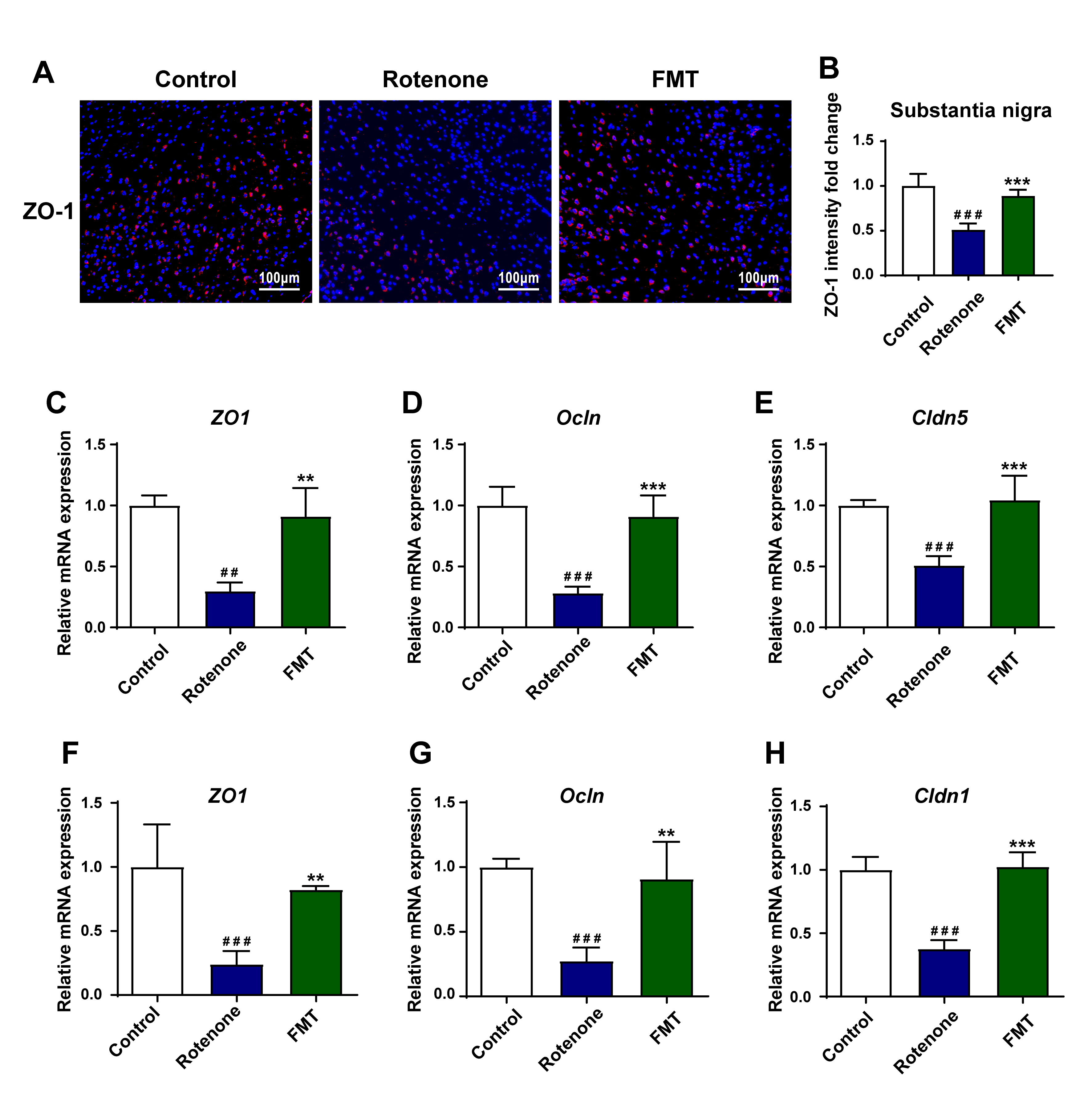

Supplement: Supplementary file 2 — Additional file 1: Fig. S1 FMT treatment alleviates the expression of tight junction proteins reduced in the rotenone-challenged mouse model. (A) Representative captures of immunofluorescence of ZO-1 in the SN. (B) The intensity analysis of ZO-1 immunofluorescence staining in the SN. (C-E) mRNA expression of tight junction proteins ZO1, Ocln and Cldn5 in the midbrain containing the SN. (F-H) mRNA expression of tight junction proteins ZO1, Ocln and Cldn1 in the colon. For (B), n = 5 for each group. For (C-H), n = 3 for each group. Data are presented as mean ± SD. ## p < 0.01, ### p < 0.001 versus the control group; ** p < 0.01, *** p < 0.001 versus the rotenone group. [file 40168_2021_1107_MOESM2_ESM.tif]

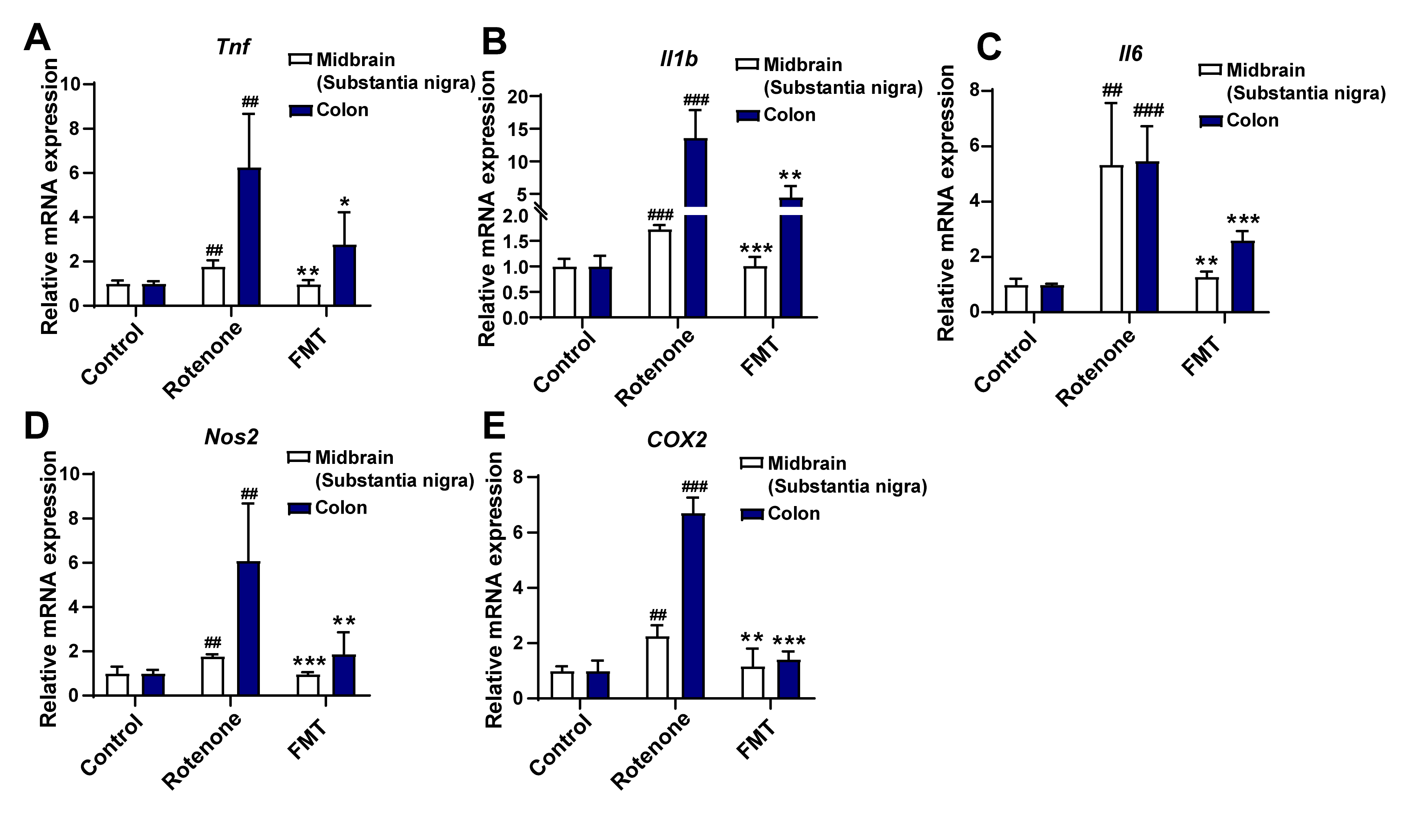

Supplement: Supplementary file 3 — Additional file 2: Fig. S2 FMT administration suppresses the generation of pro-inflammatory molecules both in the SN and the colon of rotenone-challenged mice. (A-E) mRNA expression of pro-inflammatory cytokines (Tnf, Il1b, Il6, Nos2, COX2) in the midbrain containing the SN and the colon. For (A-E), n = 3 for each group. Data are presented as mean ± SD. ## p < 0.01, ### p < 0.001 versus the control group; * p < 0.05, ** p < 0.01, *** p < 0.001 versus the rotenone group. [file 40168_2021_1107_MOESM3_ESM.tif]

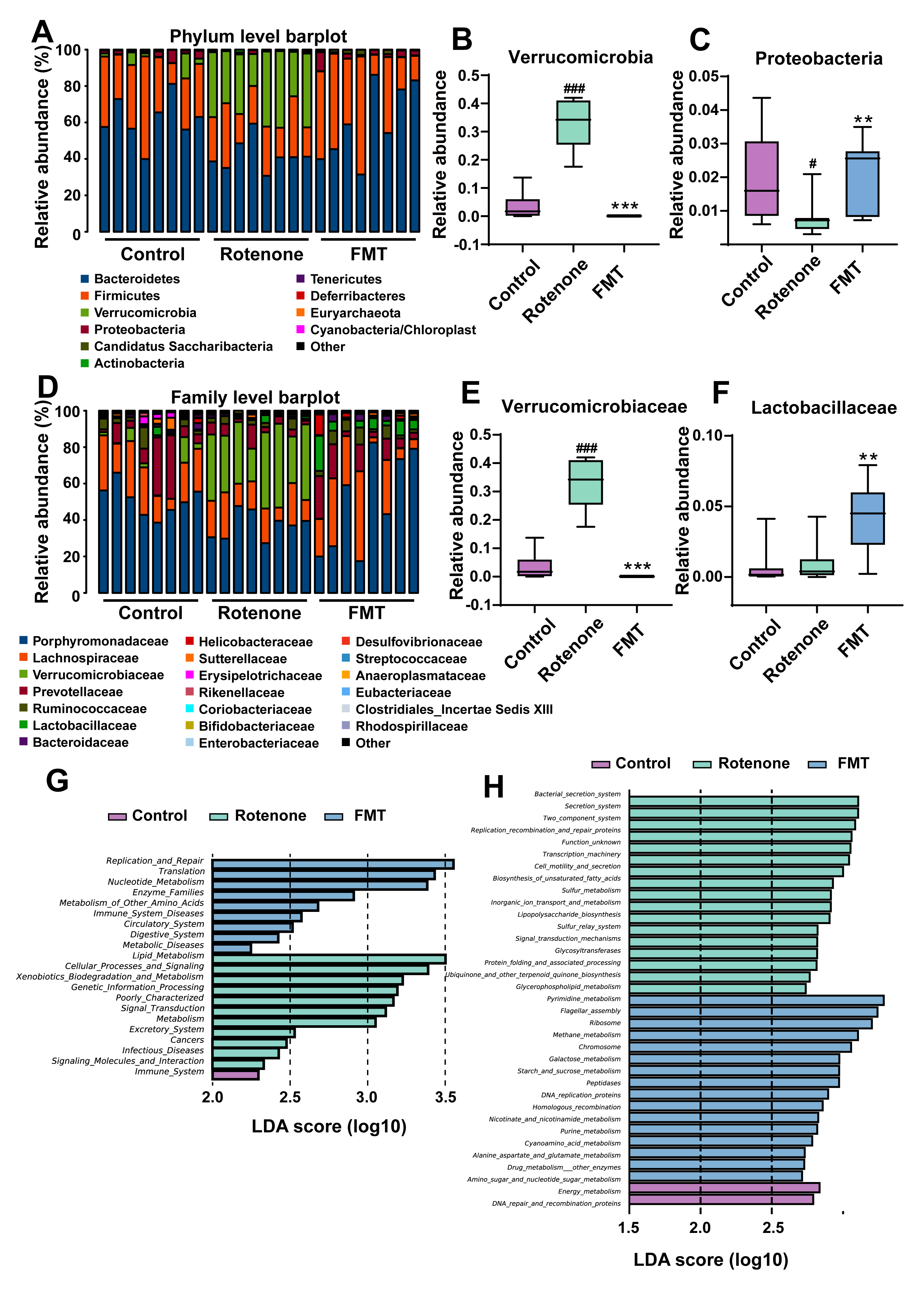

Supplement: Supplementary file 4 — Additional file 3: Fig. S3 FMT treatment attenuates microbiota dysbiosis of rotenone-intoxicated mouse model. (A) Relative abundances of gut microbiota at the phylum level in the 3 groups. (B-C) Relative abundances of significantly altered bacterial phyla: Verrucomicrobia and Proteobacteria. (D) Relative abundances of gut microbiota at the family level in the 3 groups. (E-F) Relative abundances of significantly altered bacterial families: Verrucomicrobiaceae and Lactobacillaceae. (G) Bar graph of LDA scores of enriched KEGG pathways at level 2. LDA scores (log10) > 2 and P < 0.05 are shown. (H) Bar graph of LDA scores of enriched KEGG pathways at level 3. LDA scores (log10) > 2.7 and P < 0.05 are shown. In this figure, n = 8 for each group. Each boxplot represents the median, interquartile range, minimum and maximum values. # p < 0.05, ### p < 0.001 versus the control group; ** p < 0.01, *** p < 0.001 versus the rotenone group. [file 40168_2021_1107_MOESM4_ESM.tif]
